# Extension of coarse-grained UNRES force field to treat carbon nanotubes

**DOI:** 10.1007/s00894-018-3656-1

**Published:** 2018-04-26

**Authors:** Adam K. Sieradzan, Magdalena A. Mozolewska

**Affiliations:** 1 Faculty of Chemistry, University of Gdansk, ul. Wita Stwosza 63, 80-308 Gdansk, Poland; 2 Institute of Computer Science, Polish Academy of Sciences, ul. Jana Kazimierza 5, 01-248 Warsaw, Poland

**Keywords:** Molecular dynamics, Nanotechnology, Nanotoxicity, Simulations, Single walled carbon nanotube (SWCNT)

## Abstract

Carbon nanotubes (CNTs) have recently received considerable attention because of their possible applications in various branches of nanotechnology. For their cogent application, knowledge of their interactions with biological macromolecules, especially proteins, is essential and computer simulations are very useful for such studies. Classical all-atom force fields limit simulation time scale and size of the systems significantly. Therefore, in this work, we implemented CNTs into the coarse-grained UNited RESidue (UNRES) force field. A CNT is represented as a rigid infinite-length cylinder which interacts with a protein through the Kihara potential. Energy conservation in microcanonical coarse-grained molecular dynamics simulations and temperature conservation in canonical simulations with UNRES containing the CNT component have been verified. Subsequently, studies of three proteins, bovine serum albumin (BSA), soybean peroxidase (SBP), and α-chymotrypsin (CT), with and without CNTs, were performed to examine the influence of CNTs on the structure and dynamics of these proteins. It was found that nanotubes bind to these proteins and influence their structure. Our results show that the UNRES force field can be used for further studies of CNT-protein systems with 3–4 order of magnitude larger timescale than using regular all-atom force fields.

**Electronic supplementary material:**

The online version of this article (10.1007/s00894-018-3656-1) contains supplementary material, which is available to authorized users.

**Graphical abstract:**
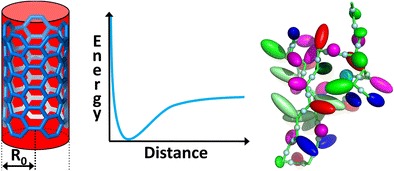
Bovine serum albumin (BSA), soybean peroxidase (SBP), and α-chymotrypsin (CT), with and without CNTsᅟ

## Introduction

Since the discovery of fullerenes and carbon nanotubes (CNTs) [1], nanotechnology has been one of the fastest developing branches in modern industry and medicine [2]. Nanotechnology is a multidisciplinary field which combines chemistry, physics, biology, medicine, engineering, and bioinformatics and it deals with the design, chemical synthesis, and implementation of super atomic-scale objects termed nanoparticles such as CNTs. CNTs can differ by size and diameter, but in general they are unique molecules, which can possess greater than 1,000,000 length-to-diameter ratios. The longest (half-meter-length) CNT was obtained by chemical vapor deposition [3]. CNTs are formed only from carbon atoms [4], but they can be functionalized with different chemical groups such as carboxylic groups, to obtain desired properties (e.g., to improve their solubility in water) [5]. Infinitely long particles (from the atomistic or protein scale point of view) are obtained in laboratories working on growing long CNTs. They can be used as very strong fiber or ballistic armors [3], or as an alternative to filaments [6].

While long CNTs are desired in industry, they can also penetrate a living organism and interact with cells and proteins [7]. Nanoparticles are also extensively investigated in the context of the delivery of potential drugs to their targets via surrounding of CNT or fullerene by ligand (corona effect) [8, 9] and their interaction with proteins and receptors. Such studies have been performed extensively both experimentally [10, 11] and theoretically [12–14]. Nanomaterials play a key role in nanomedicine and extensive research has been carried out on their use, e.g., in cancer cell treatment [15, 16].

Because of the increasing use of nanoparticles in industry, they have become present in the environment and, consequently, in living organisms. Therefore, due to frequent use of CNTs, there is a concern about their toxicity to living cells and the entire organisms [17]. In general, nanoparticles in living organisms mainly interact with proteins, and therefore studies of protein–nanoparticle interactions are very important to assess their influence on living organisms, as well as their ability to serve as drug carriers.

Interactions between a CNT and a protein can be investigated theoretically by performing simulations in either all-atom or coarse-grained force fields. One example is AutoIMD, a tool which allows simulating interactions between a CNT and a protein. That tool is implemented as an Interactive Molecular Dynamic in the popular VMD software [18]. AutoIMD enables one to manipulate the molecules during MD simulations with real-time force feedback and a graphical display. There is also a wide range of packages containing all-atom force fields which enable one (after manual parametrization) to perform the CNT simulation in the all-atom resolution (e.g., AMBER [19], GROMACS [20], and NAMD [21]). On the other hand, the systems containing a CNT and a protein can sometimes be too big to simulate at the all-atom resolution in a reasonable timescale. Therefore, coarse-grained models of the CNTs were developed. One such model was created by Chen et al. [22] using the open source LAMMPS [23] platform, where each CNT is simplified as a multi-bead chain. Another approach was developed by Wallace and Sansom [24] who considered three carbon atoms as one interaction center, and implemented it into the GROMACS package.

In this paper, we present another approach to treat protein-CNT interactions. We used the UNited RESidue (UNRES) force field with a CNT treated as a cylinder with infinite length. UNRES is a coarse-grained force field [25–27] for protein simulation, which, like other coarse-grained force fields, e.g. Martini [28], is constantly being improved and extended to treat not only proteins, but also nucleic acids, sugars, and lipids [29]. Owing to the simplified representation, UNRES provides an about 1000-fold speed-up with respect to all-atom calculations and was used in the past to investigate protein-protein interactions and to predict protein structure [30–34]. Therefore, the model developed in this study will enable us to study peptide- and protein-CNT systems at the time- and size-scale inaccessible to all-atom simulations. In other words, it will be possible to perform calculations with an off-the-shelf desktop or a laptop computer instead of a dedicated supercomputer machine such as ANTON, achieving a similar timescale [35]. Such simulations will enable us to determine the mechanisms, thermodynamics, and kinetics of interactions between the proteins and CNTs at a low computational cost and with the CNTs degrees of freedom averaged out.

## Methods

### UNRES model of proteins

**Fig. 1:**
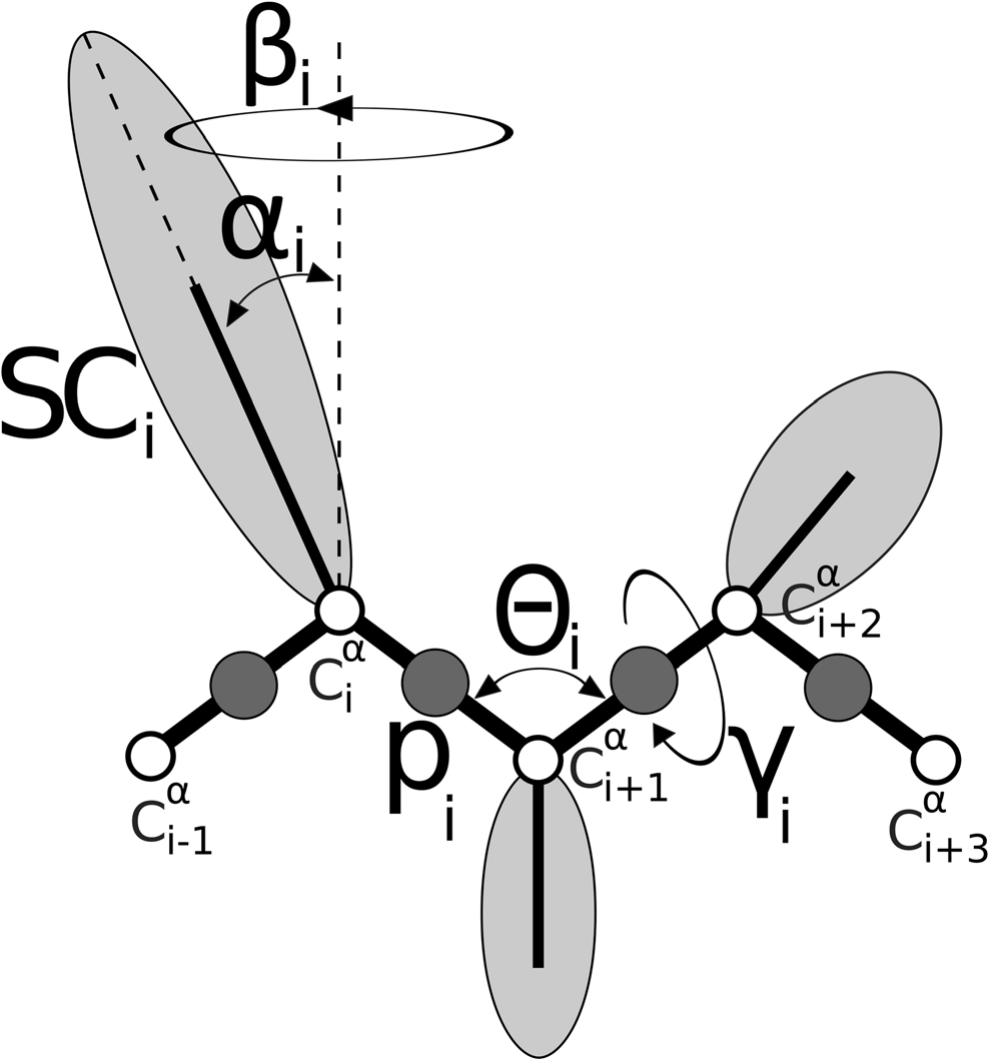
Coarse-grained UNRES model of the polypeptide chain. θ is C^α^_i_… C^α^_i + 1_… C^α^_i + 2_ virtual bond angle, γ is C^α^_i_… C^α^_i + 1_… C^α^_i + 2_… C^α^_i + 3_ dihedral angle, α and β are polar angles defining position of the side-chain. Side-chains are ellipsoids of revolution represented as light gray ellipses, peptide groups are represented by dark gray spheres (located half-way between two consecutive C^α^ which are represented by white circles)

UNRES [25–27, 36] is a physics-based coarse-grained force field designed for simulations of peptides and proteins. In this study, its applicability has been extended to protein-CNT systems. In the UNRES model (Fig. 1), each chain of the amino-acid residues is described as a series of consecutive Cα atoms (white circles) and peptide (p) groups (gray circles). The side chains (SC) are represented by ellipsoids attached to the respective Cα atoms. In the UNRES force field there are only two interaction sites per residue: i) SCs and ii) p (peptide group) centers, while Cα atoms serve only to describe the geometry of the amino-acid chain. Due to the simplification of the polypeptide chain, observed as a reduction of the number of interaction sites per amino-acid residue, the effective energy function is given as the potential of mean force (PMF), also containing multibody terms (for more details see ref. [37]). The PMF was derived from all-atom simulation of interaction centers in water (in an explicit form). However, to speed up calculations in the UNRES model, water was in implicit form (in a mean-field manner). Even though the UNRES force field has no water molecules in explicit form, it is able to fold proteins correctly [34, 38]. In the UNRES force field pH is fixed, temperature independent and set to 7, and therefore, residues Arg, Lys, Asp, and Glu are charged.

### Nanotube continuous model

**Fig. 2:**
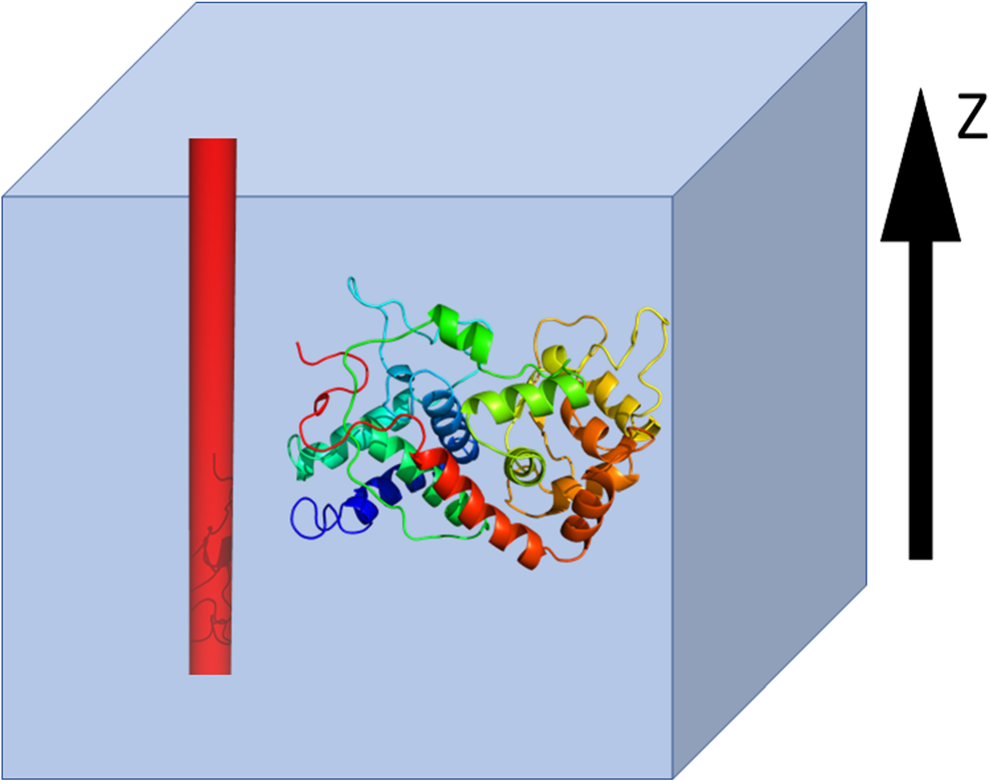
Schematic representation of the CNT-protein system in the UNRES model. The red tube whose axis coincides with the z axis, represents an infinitely-long CNT. The protein (shown as a cartoon representation) interacts only with the surface of the CNT. The system is placed in a periodic box and the tube is not subjected to translations or rotations

In our model, a nanotube is represented as an infinite cylinder with radius *R*_*0*_, whose axis coincides with the z axis and which is assumed immovable (Fig. 2).

**Fig. 3:**
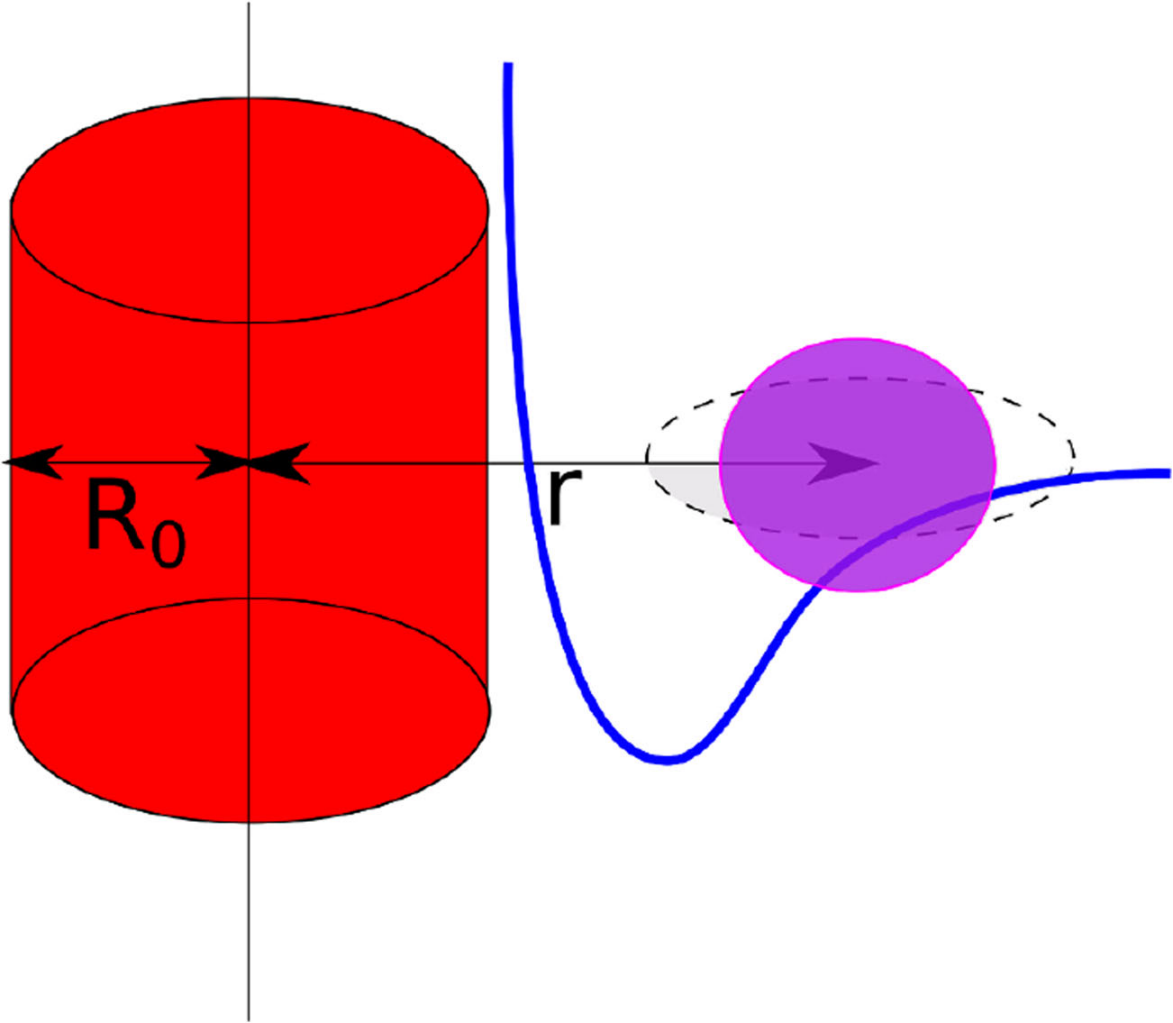
Schematic illustration of the protein-CNT interaction modeled by a modified Kihara potential, where the red tube represents the CNT and the ellipsoid is a protein interaction site (a side chain or a peptide group). It should be noted that the side-chain (gray ellipsoid) is spherically approximated (violet sphere) as the symmetrical potential is used

Consequently, simulations were performed using periodic box. As the CNT is a long rod-like object with a noncharged, hydrophobic surface, an interaction between the CNT and the protein residues (Fig. 3) is described by the extension of Lenard-Jones potential to non-spherical object, named Kihara potential [39]. This potential was modified by changing the distance from the rod like-object long axis to the surface and is expressed by Eq. 1.

1 $$ {U}_{CNT}=4\varepsilon \left[{\left(\frac{\sigma }{r-{R}_0}\right)}^{12}-{\left(\frac{\sigma }{r-{R}_0}\right)}^6\right] $$

where ε - potential well depth, σ - distance where the potential is equal to 0, r - distance between the center of interaction and nanotube axis, r-R_0_ - distance between the surface of the CNT and the interacting center. It should be noted that in the UNRES force field side-chains are represented by spheroids; however, a spherical approximation was used for protein-CNT interactions. This approximation is used as the small side-chain interaction with large CNT surface, and should not be applied when interacting centers are of similar size. Because the CNT is composed of carbon atoms, the parameters for protein–side-chain interactions with the CNT were adapted from the interaction of the phenylalanine side-chain with other interaction centers, while peptide group-CNT interaction was approximated as a glycine-phenylanaline interaction. The complete energy function is expressed by Eq. 2.

2 $$ {U}_{CNT- protein}={U}_{UNRES}+{w}_{CNT}{U}_{CNT} $$

where *w*_*CNT*_ is the weight of the new CNT potential. Optimization of the w_CNT_ weight is extremely challenging since it requires both experimental NMR data of CNT with proteins at different temperature ranges and use of the maximum likelihood method applied to a series of simulations. Therefore, in the current work w_CNT_ was arbitrarily set to 1.

### Test of the CNT potential

#### Microcanonical simulations of CNT potentials

In UNRES, the conformational search is carried out by using coarse-grained molecular dynamics and its extensions. Therefore, after implementing the nanotube in the UNRES, we checked energy conservation in the constant volume, constant number of particles, and constant energy (NVE) MD simulations also termed microcanonical simulations. We used the Trp-cage (PDB ID: 1L2Y) as a model system. Initially, the protein was placed inside of the periodic box with each of the sides equal to 220 Å. The first residue was placed ~10 Å from the CNT center with a CNT radius (R_0_) of 6 Å resulting in a slightly positive value of the protein-CNT interactions. This structure was energy minimized with the energy weight *w*_*CNT*_ = 1; the same weight was used in all further investigations. An energy-minimized approximately 400 ns (or approximately 400 μs real time after taking into account 1000 times speed-up of the UNRES force field; the speed up is a result of lowering the barrier height, the omission of low degrees of freedom and changing degrees of pathway along which the reaction occurs [40]). Microcanonical molecular dynamics simulations with the variable time step (VTS) integrator was carried out [41, 42].

#### Influence of the CNT on thermostat behavior

To study the influence of the newly introduced CNT on thermostat behavior, canonical simulations with the Langevin, Berendsen, and Nose-Hover thermostats [41, 43–45] were carried out. The simulations were performed at 200, 300, 400, and 500 K. For the Berendsen thermostat [43], coupling parameter τ = 48.9 fs was used. To speed up the calculation in Langevin dynamics, the factor of 0.01 for water friction was used [41]. For Nose-Hoover thermostat, the “thermostat effective momentum of inertia,” Q [45] was set at 5 kcal/MTU^2^/mol. The length of the simulation was 5 ns (corresponding to ~5 μs of real time) with a time step of 0.489 fs.

### MREMD simulations

To test the implementation of the CNT model and the relevance of the CNT-protein interaction potential as developed in this work, three proteins were chosen: bovine serum albumin (BSA), soybean peroxidase (SBP), and α-chymotrypsin (CT); (PDB IDs: 1FHF, 1EX3, 4F5S, respectively). The two latter proteins originate from bovine pancreas. As a reference, a series of multiplexed replica exchange molecular dynamics (MREMD) simulations without CNT were performed. REMD is a technique in which multiple trajectories at a wide range of temperatures are run, and trajectories can exchange between temperatures based on the Metropolis criterion after a given number of MD steps. MREMD is an extension of the REMD method, in which multiple trajectories at a given temperature are run to improve scalability [25, 46, 47]. Such an approach significantly improves the conformation-space search performed by the UNRES implementation up to 75% scalability on 4096 CPUs.

Subsequently, simulations were carried out using the UNRES coarse-grained force field with the implemented continuous model of the CNT. All simulations with and without nanotube were performed with the Langevin thermostat and the variable time step (VTS) algorithm [25, 41] at 36 temperatures: in the range 250–370 K temperatures were sampled every 5 K steps, in 370–460 K range every 10 K, and in 460–500 range every 20 K; two trajectories per temperature were simulated, which gives a total of 72 trajectories. Reference simulations without CNTs and simulations with CNTs of various diameters: 4 Å, 4.5 Å, 5 Å, and 5.5 Å for SBP and CT (similarly as in earlier work [48]), and 8.85 Å, 11.1 Å, and 13.3 Å for BSA (similarly as in earlier work [49]) were performed. Therefore, SBP and CT were analyzed together and BSA was analyzed separately.

Additionally, a series of MREMD simulations with weak, structure-based Lorentzian-like restraints imposed on the protein under study were performed. The flat bottom Lorentzian-like restrains [50] on Cα atoms were used because of the large size of the systems, length of the simulations, and to ensure high stability of structures of the proteins (Eq. 3). [51]

3 $$ U=\left\{\begin{array}{c}0\kern1em if\;{x}_0-0.1<x<x+0.1\\ {}\frac{A{\left(x-{x}_0+0.1\right)}^4}{{\left(x-{x}_0+0.1\right)}^4+\sigma } if\;x\le {x}_0-0.1\\ {}\frac{A{\left(x-{x}_0-0.1\right)}^4}{{\left(x-{x}_0-0.1\right)}^4+\sigma } if\;x\ge {x}_0+0.1\end{array}\right. $$

where *x*_0_ is a reference (native-structure) distance between the sites which are in contact in the native structure, *x* is the current distance between interacting sites, *A* is a scaling factor set to $24/{x}_0^2$, and *σ* was set to (0.04*x*_0_)^2^, as in our earlier work [52]. The *A* and *σ* values and the relative weight (*w*_*restraint*_ *= 0.006*) were derived to obtain ~2.5 Å RMSD fluctuations from the native structure in the simulations carried out for SBP without CNT at 300 K.

Each trajectory for SBP, CT, and BSA consisted of 57 million steps, where each step is 4.89 fs, which gives about 285 ns of UNRES time, and about 285 μs of real time. As a starting structure for the simulation, the native structures of SBP, CT, and BSA proteins were used after short energy minimization. For all simulations with CNTs, the initial distance between the CNT surface and the closest residue of each of the proteins was set to 8 Å. The last, equilibrated part of the simulations, corresponding to 280–285 μs of real time was used in further analysis.

Therefore, for each protein four different simulation approaches were used:

- unrestrained protein without CNT,
- restrained protein without CNT,
- unrestrained protein with CNT,
- restrained protein with CNT.

For clarity, a detailed description and figures for unrestricted simulations are provided in the SI.

After MREMD simulations the weighted histogram analysis method (WHAM) [53–55] was applied to determine the heat-capacity (Eq. 4) [25] profiles and conformational ensembles at any desired temperatures.

4 $$ {C}_{\nu}\left(T,\left\{U\right\},\left\{X\right\}=-{\left\langle T\frac{\delta^2U\left(X,T\right)}{\delta {T}^2}\right\rangle}_T+\frac{1}{RT^2}{\left\langle \left\langle {\left[U\left(X,T\right)-T\frac{\delta U\left(X,T\right)}{\delta T}\right]}^2\right\rangle \right\rangle}_T\right) $$

The heat capacity is a very informative feature. The heat capacity peak temperature occurs at the transition from unfolded to folded state. If there is a single narrow peak, it means that the process involves cooperative conformational changes on multiple protein fragments. If the peak is broad or multiple peaks occur, it indicates that the process is less cooperative and multiple transition states are present. The higher the heat capacity peak is, the larger the energy changes present during conformation changes. A peak present in the high temperature region means that the protein is very stable thermally [25]. It should be noted that introduction of artificial stabilizing restraints leads to overstabilization of the protein and a shift of the heat capacity peak to higher values; it also may lead to more cooperativity occurring during protein (un)folding.

Afterward, cluster analysis was performed using Ward’s Minimum Variance Method [53–55], with RMSD as the measure of the distances between the conformations. To perform comparative clustering, we first performed cluster analysis of the simulated conformational ensembles for each protein without the CNT to obtain exactly ten clusters. The RMSD cut-off values obtained in this way, were used in the analysis of each protein-CNT simulation. Additionally, average fractions of contacts were calculated as the number of the Cα atoms within 8 Å from the CNT surface divided by the number of residues averaged over all structures clustered.

Additionally, the average probability of contact for a given residue was calculated with the use of Eq. 5:

5 $$ p(x)=\frac{\sum {N}_{xdist<8}}{\sum N} $$

where *N*_*xdist* < 8_ is the number structure where x Cα atom is within 8 Å from the CNT surface divided and N is the number of residues averaged over all structures clustered.

## Results and discussion

### Microcanonical simulations of the tryptophan cage-CNT system

**Fig. 4:**
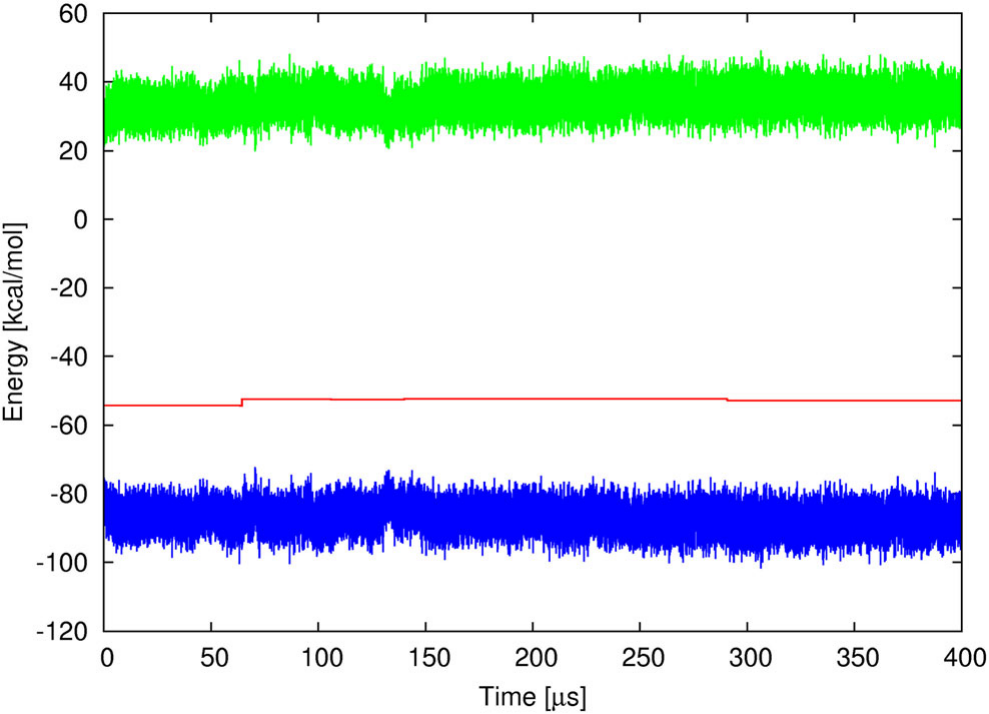
Plots of the total (red), potential (blue), and kinetic (green) energy for microcanonical simulation of Trp-cage miniprotein – CNT system

A plot of the kinetic, potential, and total energy vs. time is shown in Fig. 4. As shown in the figure, the total energy is fairly constant and its fluctuations are significantly smaller than the kinetic energy and the potential energy fluctuations. The total energy fluctuation over 400 μs (400 ns UNRES time) simulation is less than 1.5 kcal mol^-1^ and no systematic energy drift can be observed; only a small energy jump occurs at about 60 μs. This indicates that, despite the introduction of a nanotube, the total energy is kept by the system and no occurrence of artificial force is observed even in a relatively long run. Consequently, the extended UNRES model can be used to simulate the CNT-protein systems without any energy artifacts.

### Influence of the CNT potential on CNT-protein system temperature

Since the introduction of the nanotube model could disturb the temperature controlling algorithms, we analyzed the average temperatures of the Trp-cage – CNT system maintained by three thermostats implemented in UNRES (Table 1). As can be seen, there is no overheating effect for Nose-Hoover or Berendsen thermostats but, clearly, the average temperature is slightly overestimated with the Langevin thermostat. As described in an earlier work [56], this effect is an artifact of using periodic boundary conditions (PBC). When linear equation *f(T) = aT* is fitted, the overestimation coefficient *a* is equal to 1.0046, and therefore, the error is smaller than 0.5% and can be neglected. Because the CNT degrees of freedom are removed, the average temperature should be overestimated to a lesser degree than without CNT [48]; however, this was not observed (Table 1). It suggests that the introduction of CNT does not influence the thermostat behavior to any degree.

**Table 1:** Average temperature of Trp-cage – CNT system maintained by a given thermostat obtained during simulation compared with the set thermostat temperature

Thermostat type				
Set temperature [K]	Berendsen	Nose-Hoover	Langevin	Langevin w/o CNT
200.000	199.820	200.891	201.057	200.707
300.000	299.930	300.935	301.452	300.936
400.000	399.802	400.366	402.081	401.378
500.000	499.969	500.658	502.271	502.892

**Fig. 5:**
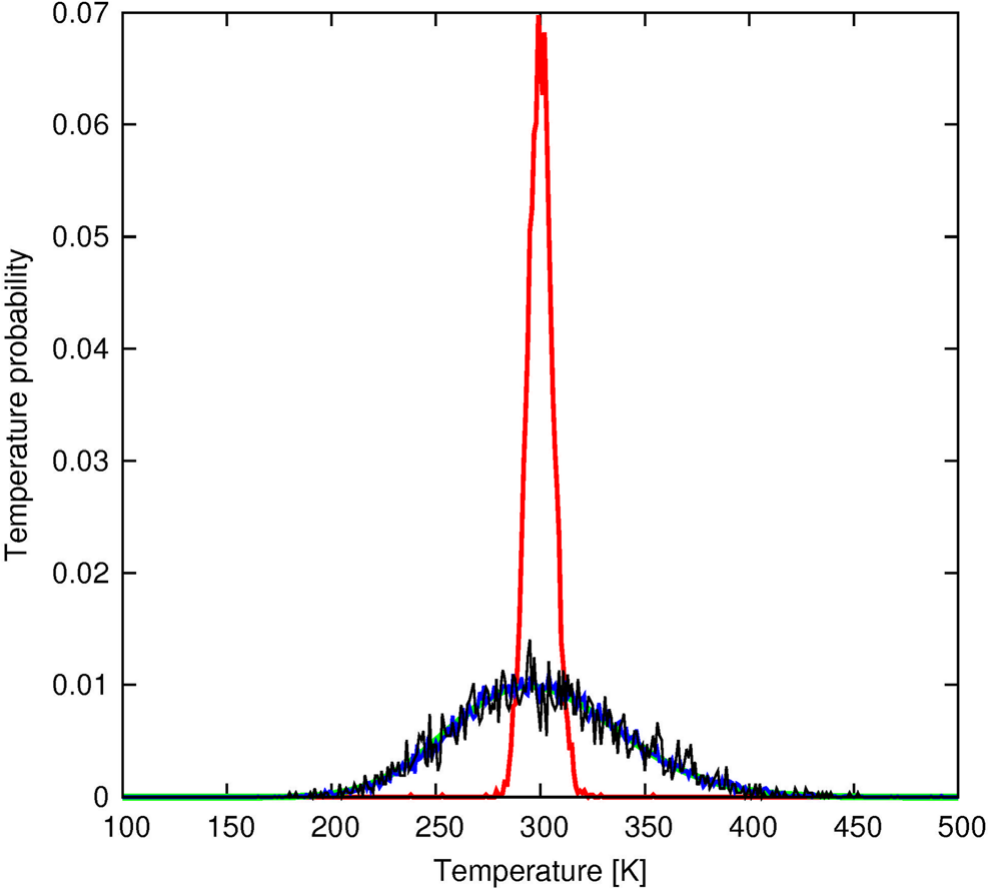
Temperature distribution plots for the Berendsen (red), Langevin (blue), and Nose-Hoover (black) thermostats (*T* = 300 K) compared with the theoretical distribution (green) for the Trp-cage – CNT system

The Berendsen thermostat produced too narrow a temperature distribution (Fig. 5) which is known from literature but was verified, since the thermostat is frequently used in the UNRES force field. Both the Nose-Hoover and Langevin thermostats produced correct temperature distributions; however, it should be noted that, for the Nose-Hoover thermostat, the Q parameter is system-specific and should be adjusted to keep the correct distribution.

### Protein-CNT MREMD simulation results

#### CT with restraints

For the CT protein in simulations without CNT, the protein is stable (RMSD <4 Å), and both RMSD and the RG does not change significantly up to ~405 K (Fig. S1b and S2b). With the CNT present in the system, RMSD and RG start to increase at much lower temperature: 350 K and 360 K, respectively, leading to complete unfolding above 390 K.

The heat capacity (CV) peak for the protein without CNT occurs at 405 K; while with the CNT it shifts down to 393 K (Fig. S3b). During the simulation, the protein had big mobility and moved around the CNT (Fig. S1c and d). The populations of the families within the cluster for the simulation decreased (Table S1) in comparison to simulation without CNT (Table S2), which indicates that presence of the CNT stimulates conformations changes of the CT. The impact of the CNT on the CT structure increases with the decrease of the CNT diameter; however, the difference is rather small.

#### SBP with restraints

For simulations of the SBP protein without CNT but with restraints, the RG increases very slowly in the whole temperature range, and its temperature dependence is quite similar to that of the simulation without any restraints. With the CNT, the behavior is similar up to ~365 K, but above that temperature RG starts to increase rapidly; however, the increase is slower than that for the analogous CT simulations. The difference of RG between the simulations with and without CNT is around 5 Å at 380 K. The RMSD value for the protein without CNT does not change until 400 K, and then a significant increase is observed, while with CNT, RMSD starts to increase at 375 K; however, as for RG, the increase is slower than that for the CT protein. For the SBP protein simulated without CNT, the heat-capacity peak occurs at 405 K, while in the simulations with CNT it occurs at 385 K (Fig. S3d); however, the peak heat-capacity values are 3–4 times smaller than those for the CT protein. All these observations indicate that interactions with CNT influence the structure and stability of CT more strongly than those of SBP.

#### Comparison of CT and SBP simulations

As mentioned in the preceding section, for the SBP protein, the heat capacity band calculated from restrained simulations is shifted to higher temperatures and is narrower compared to that resulting from unrestrained simulations, the latter heat capacity is also multimodal. It can be seen from Fig. S2d that, as for the CT protein, the ensemble-averaged radius of gyration remains almost independent of temperature in the simulation without, whereas it varies significantly with temperature in simulation with CNT. However, the change is not as big and drastic as that for the CT protein. The population of conformations within the family for the simulation with restraints increases significantly (Table S3) in comparison to simulation without CNT for both temperatures 290 K and 300 K (Table S4).

The RMSD vs. temperature plots for both the SBP and the CT protein (Fig. S2) show that the proteins change their structure and the RMSD without CNT and with CNT increases. With CNT for SBP this increase is significant; however, RMSD changes are not as big as for the CT protein. This indicates that the CT protein loses its structure more readily and, thereby, its catalytic activity changes at room temperature upon interaction with nanotube; the SBP protein changes its structure only slightly and is, therefore, unlikely to lose its catalytic activity (Fig. S1 g and h).

Also, the cluster analysis indicates higher stability (Table S1-S3) of the SBP protein interacting with the CNT than in the case of the CT protein. When comparing the dominating cluster at 300 K, the average RMSDs increase, which is caused by the presence of the CNT for simulations with restraints, is 0.7 Å and 0.4 Å for CT and SBP, respectively. For simulations with restraints, both proteins exhibit a similar fraction of residues in contact with the CNT and a similar range of binding energy (Table 2), the CT having a slightly higher fraction of residues in contact with CNT and a slightly higher binding energy, on average. However, when the simulations without restraints are analyzed (Table 2), a different pattern emerges. The CT protein binds to CNT significantly more strongly than the SBP protein. It is worth mentioning that, for SBP and CT in simulations without restraints, the presence of the CNT results in the same or increased diversity of structures as the number of clusters increases (Table S1 and S3). It should be noted, that because of the use of a coarse-grained force field, only relative values should be analyzed. Large absolute values are the result of using unmodified CNT, which forms very strong hydrophobic interactions with the proteins (that are especially visible for the CT protein, whose structure was deformed to maximize contacts between hydrophobic residues and the CNT (Fig. S1c)).

**Table 2:** Average fraction of contacts and average binding free energy (kcal mol^-1^) for the protein-CNT systems studied in this work at 300 K for simulations with and without restraints

		With restraints		Without restraints	
Protein	CNT radius	Average contact fraction	Binding energy	Average contact fraction	Binding energy
CT	4.0 Å	0.13	70.8	0.44	237.6
	4.5 Å	0.13	68.8	0.40	219.7
	5.0 Å	0.12	69.3	0.38	208.8
	5.5 Å	0.11	73.3	0.35	217.9
	Average	0.123	70.55	0.393	221.0
SBP	4.0 Å	0.10	65.8	0.25	134.7
	4.5 Å	0.11	67.4	0.17	124.6
	5.0 Å	0.13	79.0	0.40	171.1
	5.5 Å	0.10	65.9	0.23	164.0
	Average	0.110	69.53	0.263	148.6
BSA	8.85 Å	0.04	124.8	0.12	305.6
	11.1 Å	0.07	96.1	0.13	160.9
	13.3 Å	0.08	118.5	0.08	118.2
	Average	0.063	113.1	0.11	194.9

#### Binding frequency analysis

**Fig. 6:**
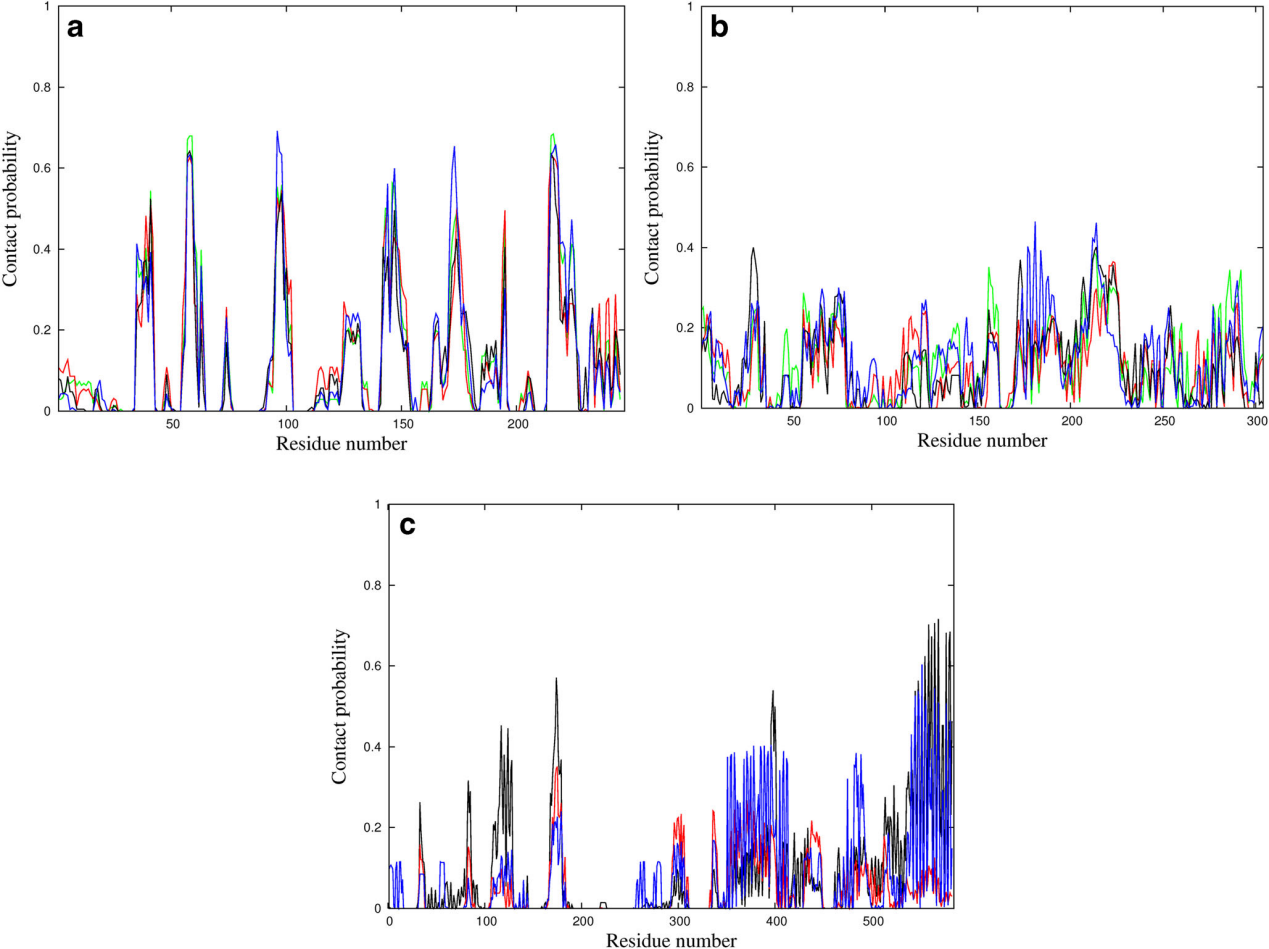
The probability of the residue to form a contact with CNT against residue number (Eq. 5) for simulations with weak restraints of: a) CT protein with CNT diameters of 4.0 Å (red), 4.5 Å (green), 5.0 Å (blue), and 5.5 Å (black); b) SBP protein with CNT diameters of 4.0 Å (red), 4.5 Å (green), 5.0 Å (blue), and 5.5 Å (black); c) BSA protein with CNT diameters of 8.85 Å (red), 11.1 Å (blue), and 13.0 Å (black)

When a binding frequency residue by residue (Fig. 6 and S4) is analyzed, a clear pattern for the CT protein is revealed. Despite the fact that the patterns with and without restrains for CT are different, they present high frequency of a contact formation near a catalytic triad (Ser^195^, His^57^, Asp^102^) [57].

**Fig. 7:**
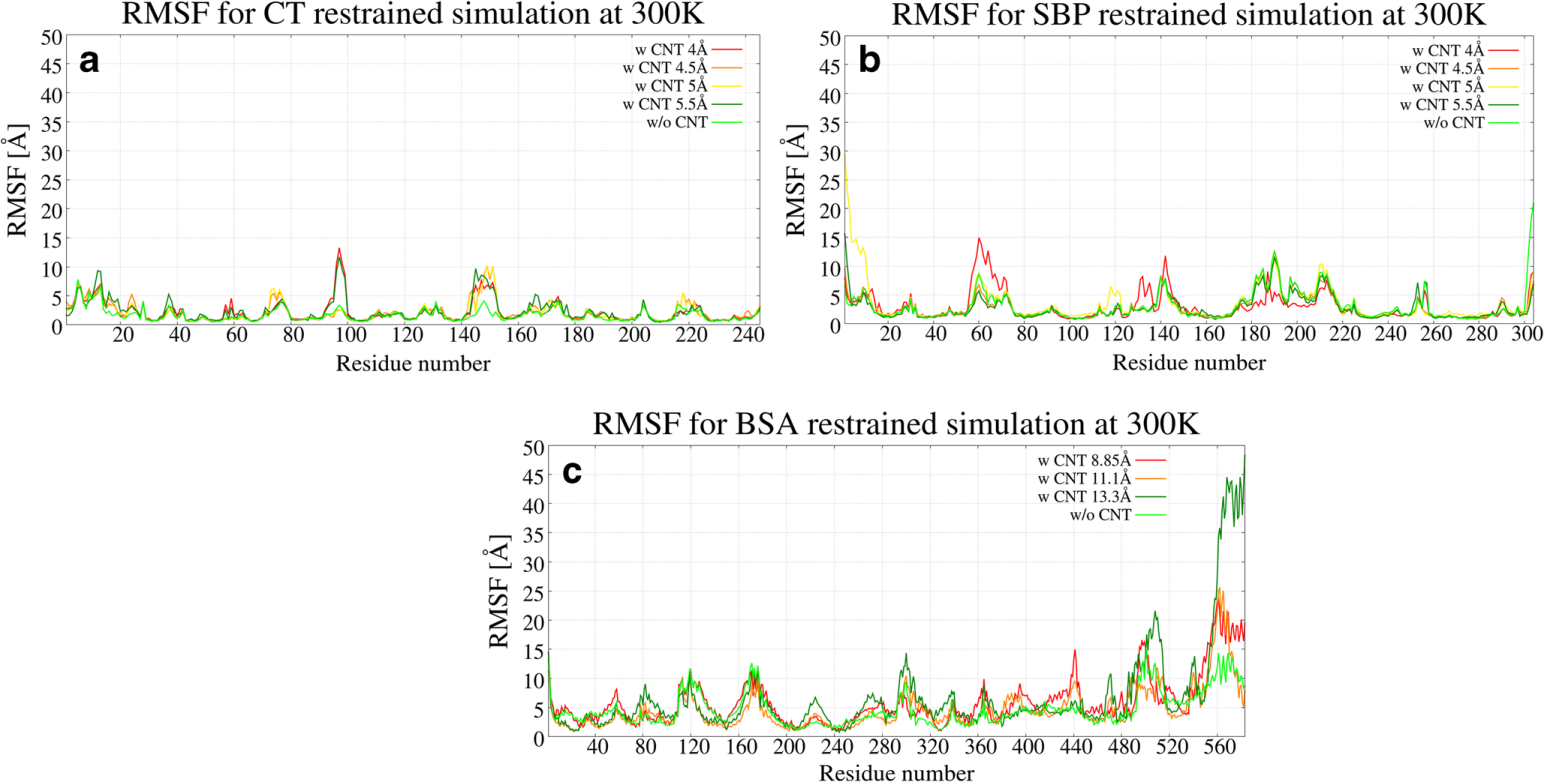
The RMSF plots obtained from the dominant cluster for CT in restrained simulations at 300 K for CT (a), SBP (b), and BSA (c)

Moreover, the pattern occurs within one type of simulation (with or without restraints) and is preserved among different CNT diameters. This indicates that the CT protein seems to bind specifically (targeted) to CNT and also suggests that the CNTs can act as CT inhibitors, which is known from literature for other enzymes [58]. This is also confirmed by Fig. 7, in which residue-wise deviations from the native structure are plotted. It can be observed that the most significant deformations occur in regions interacting with the CNT. Two such regions can be identified: 1) Ser^92^, Ser^96^, Leu^97^, Thr^98^, and Asn^100^; 2) Gly^142^, Thr^144^, Tyr^146^, Thr^147^, Asn^148^, Ala^149^, and Asn^150^, which are close to the catalytic center. On the other hand, for the SBP, the pattern is more complex and not similar among different sizes of the CNT (Fig. 6 and S4); thus, indicating that the SBP protein is binding non-specifically. Despite the non-specific binding pattern, the deformation plot (RMSF (root-mean-square-fluctuation), Fig. 7 and S5) reveals that regions Thr^60^, Asn^216^, and Leu^217^ are most readily distorted by the presence of the CNT [48]. Those residues are close to the biologically relevant hydrophobic pocket.

#### BSA with restraints

The heat capacity peak in simulation with restraints when CNT is absent occurs at around 435 K, while when CNT is present, it occurs at around 415 K (Fig. S3). With the CNT present, the protein stability is decreased. The radius of gyration for this protein with the CNT present changes rapidly with temperature starting from 400 K, while without CNT the protein is stable. The RMSD plot suggests that, when the CNT is absent, the protein is stable and only loses stability at higher temperatures (from 430 K), while when CNT is present, the RMSD changes significantly (from 405 K), which indicates that the protein changes its structure. It is worth mentioning that, for the simulations without restraints, the number of non-native clusters increases significantly at 290 K and 300 K (Table S6), which suggests that the protein loses its stability and secondary structure. The number of conformations within a family for the simulation with restraints does not change significantly in comparison to the simulation without CNT for 290 K and 300 K (Table S5).

**Fig. 8:**
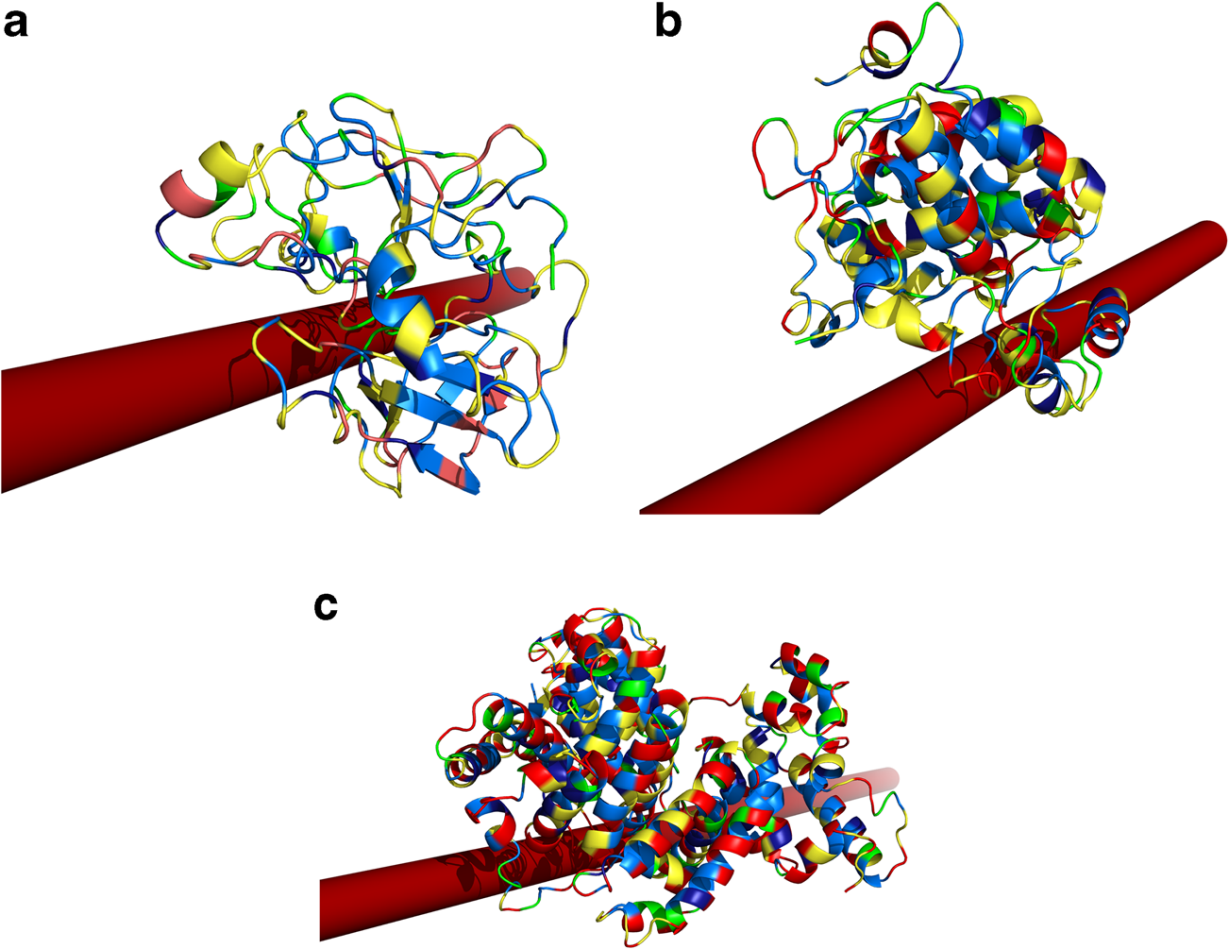
Cartoon representations of the most probable cluster structure with CNT diameter of 4 Å for 1EX3 (a) and SBP (b), and 8 Å for BSA (c) from restrained simulations. Different colors of proteins indicate the amino-acid residue types: hydrophobic (light blue), aromatic (dark blue), polar (yellow), charged (red), cysteine, proline, and glycine (green)

For BSA, the smallest fraction is bound to the CNT (Table 2); however, this binding is the strongest, which is reasonable because the BSA is the largest protein studied in this paper. Moreover, when the binding energy is compared with that corresponding to the modified multi-walled nanotube [59], the UNRES force field appears to produce too high a binding energy. This can be explained by higher hydrophobicity of the CNT studied in this paper than that studied experimentally as well as not an exact description of CNT behavior. Nevertheless, the binding energies obtained from all-atom simulations of protein binding to SWCNT [49] are in good agreement with energies obtained from our simulations (Table 2). The structure of the BSA protein with nanotube are presented in Fig. 8c.

The residue binding pattern (Fig. 6) reveals that the protein-CNT binding patterns depend on the CNT radius. For simulation with restraints, the C-terminal fragment is mostly involved in binding. For restraint-free simulations, all residues are involved in CNT binding. All these observations suggest that binding CNT to the BSA protein is non-specific.

Zhao et al. [59] performed spectroscopy studies from which they also concluded that the BSA binding to CNT is non-specific. However, all-atom simulations performed by Zu el al. [49] showed that a large C-terminal fragment seems to bind slightly more frequently. This result is similar to our theoretical predictions. Moreover, in agreement with our study (Table 2), the fluorescence spectroscopy data reported by Zu et al. [49] showed that, with a bigger CNT diameter, the number of contacts increases. This observation also suggests that the BSA protein binds nonspecifically to the CNT, which is also in agreement with the experimental data [59].

## Conclusions

We implemented a model of the infinitely long CNTs in the coarse-grained UNRES model of proteins. The sidechain-nanotube interactions were assumed to be side-chain dependent. The phenylalanine side chain with other amino acid potentials served as a basis to derive the parameters for the CNT. We proved that the total energy is conserved in microcanonical simulations of a system composed of a protein and a nanotube and the set temperature is kept in canonical simulations with the Berendsen, Nose-Hoover, and Langevin thermostats.

The test simulations performed for three example proteins, CT, SBP, and BSA, showed that CT and BSA are more susceptible to the presence of a CNT, regardless of whether the structure of the protein under consideration is restrained or not. Such observation is in agreement with the experimental data [48, 59], which indicates that the CT protein loses its catalytic activity upon interactions with the CNTs because of significant structural changes following the binding, while the nanotube binding does not affect the structure of the SBP to such a large extent; thus, allowing the protein to maintain its catalytic activity. Thanks to the extensive conformational search of MREMD simulations in the UNRES force field, our final results are reliable and do not depend on the starting position of the protein with respect to the CNT.

Analysis of the binding patterns reveals that the SBP and the BSA proteins bind to the CNT non-specifically, whereas the CT protein binds specifically near the catalytic region. Our study suggests that the CNT can act as an enzyme inhibitor, as claimed in the literature for other proteins [60]. Consequently, after further optimizations of the *w*_*CNT*_ and the UNRES force field itself, our method can find applications in nanomedicine, including design of cancer therapies and drug transport to the target cells and other studies of protein-CNT interactions, with possible extension to the DNA-CNT systems.

The UNRES software is publicly available at www.unres.pl. The carbon nanotube extension is available in “AFM” branch of a git repository of UNRES package: http://mmka.chem.univ.gda.pl/repo/unres.git.

## Electronic supplementary material

ESM 1 (DOCX 6322 kb)
