# Supplementary material for: Extension of coarse-grained UNRES force field to treat carbon nanotubes
Source: J Mol Model. 2018 Apr 26;24(5):121. doi: 10.1007/s00894-018-3656-1 (PMC5920012; doi:10.1007/s00894-018-3656-1)
Supplement: Supplementary file 1 — (DOCX 6322 kb) [file 894_2018_3656_MOESM1_ESM.docx]

Supplementary information

Introduction of Carbon Nanotubes into Coarse-Grained UNRES Force Field

Adam K. Sieradzan^1^, Magdalena A. Mozolewska^1,2*^

^1^Faculty of Chemistry, University of Gdansk, ul. Wita Stwosza 63, 80-308 Gdansk, Poland

^2^Institute of Computer Science, Polish Academy of Sciences, ul. Jana Kazimierza 5, Warsaw 01-248, Poland

Corresponding author: phone: +48 22 380 05 98, email: [m.mozolewska@ipipan.waw.pl](mailto:m.mozolewska@ipipan.waw.pl)

- - 1. *CT without restraints (simulation type 1 and simulation type 3)*

It follows in the simulations carried out for the CT-CNT systems without restraints, the protein changes its structure even at low temperatures (FigureS1-S3). The radius of gyration (RG) of the protein without CNT increases very slowly while, for the CNT-protein systems, its increase is much faster. The difference of RG between the simulations with and without CNT is up 10 Å at 300K. When the CT interacts with a CNT, the first significant increase in the RG is observed at 280K, then the flattening of the plot is observed (Figure S1a) giving a sigmoidal shape of the plot within the 260-320 K temperature range. Then, at 330 K, the RG starts again to increase rapidly. The root-mean-square deviation (RMSD) for the protein alone continues to increase to 300K and stabilizes above this temperature. For simulation with the CNT, RMSD increases constantly, except for a small plateau from 295 K to 320 K. The RMSD difference between the simulation with CNT and without CNT is ~5Å at 290-310K and increases rapidly for higher temperatures. The main heat capacity (CV) peak occurs at ~290K and it is multimodal (Figure S3a).

- - 1. *SBP without restraints (simulation type 1 and simulation type 3)*

For SBP protein in simulations without restraints, the protein without CNT (simulation type 1) changes its structure, but the RG value increases very slowly. On the contrary, the RG value for the protein with CNT (simulation type 3) increases rapidly starting from 340K. The difference of the RG between the simulation without CNT and that with CNT is around 7Å at 300K, which is by 3Å smaller than for CT. The RMSD with and without CNT is similar at 300K and varies from 0 to 5Å at 290-345K temperature range, which is significantly smaller than the 5-12Å for the CT in the same temperature range. For the protein without CNT and restraints (simulation type 1), the RMSD value changes up to 320K and is stable at higher temperatures. The RMSD for the simulations with CNT (simulation type 3) increases rapidly starting from 340K. The heat capacity band occurs in range from 270K to 350K (Figure S3c).

- - 1. *BSA without restraints*

For the BSA protein simulated without restraints, the heat-capacity peaks are in the temperature region of 260K to 350K (Figure S3). The presence of the CNT results in an upshift of the heat-capacity band to the region from 330K to 350K. However, when the CNT is not present, the high temperature heat capacity band disappears. The RG value for the simulation without CNT and without restraints does not change significantly throughout the whole temperature range (Figure S1e). For the simulation with CNT and without restraints, RG starts to change nonlinearly at 280K and grows rapidly from 320K. The difference of RMSD between the simulations is around 5Å at 300K. The RMSD for the protein without CNT and without restraints is high and the protein is unfolding. The RMSD value for the protein with CNT but without restraints is rapidly increasing from 320K.


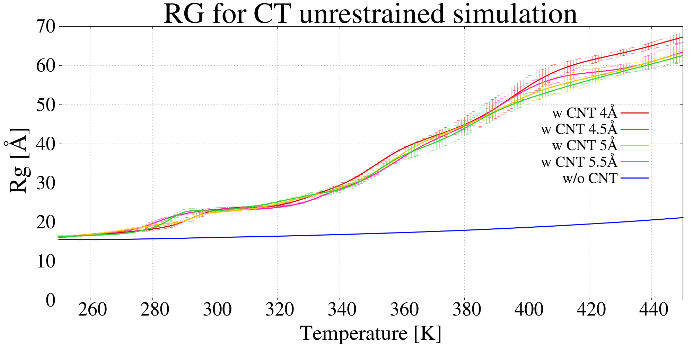
a
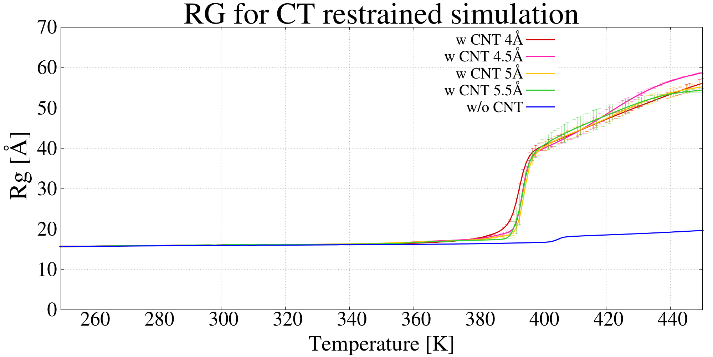
b


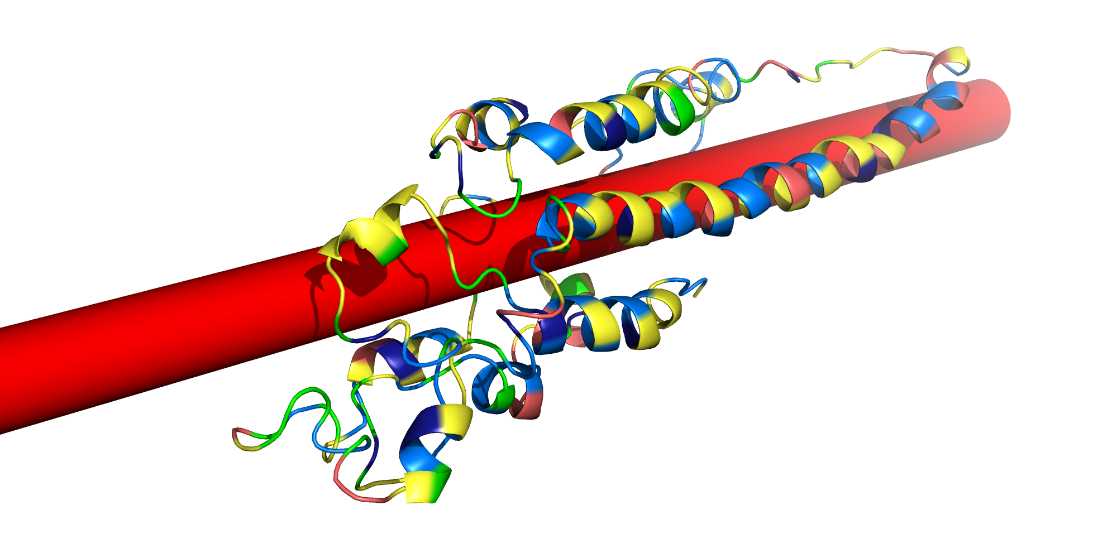
c
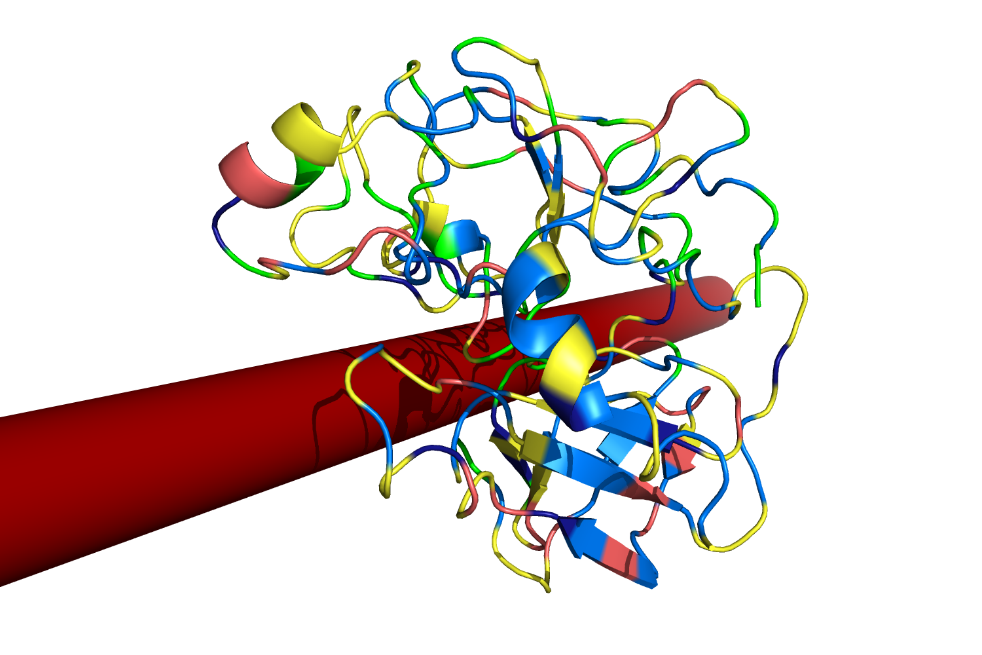
d


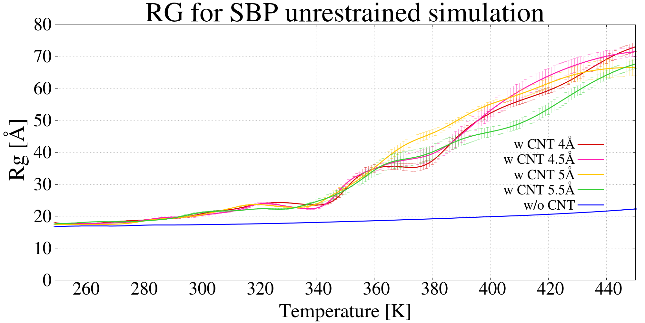
e
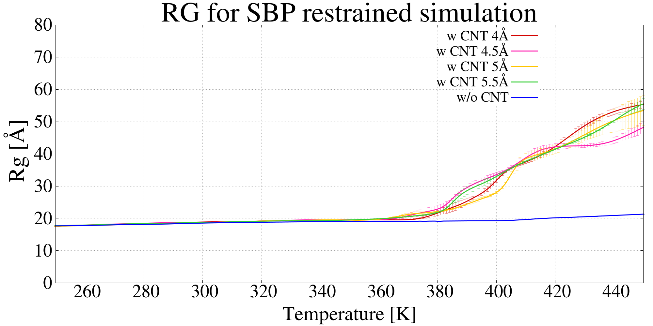
f


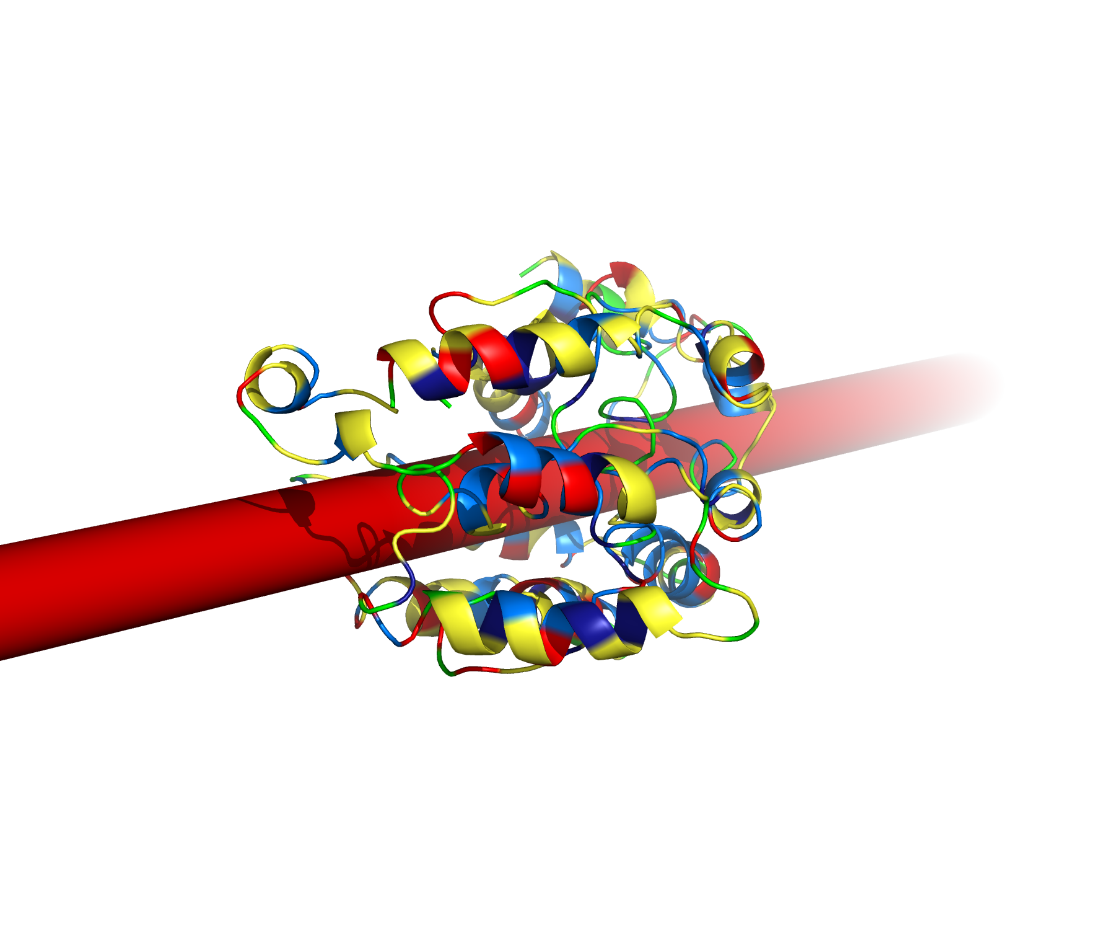
g
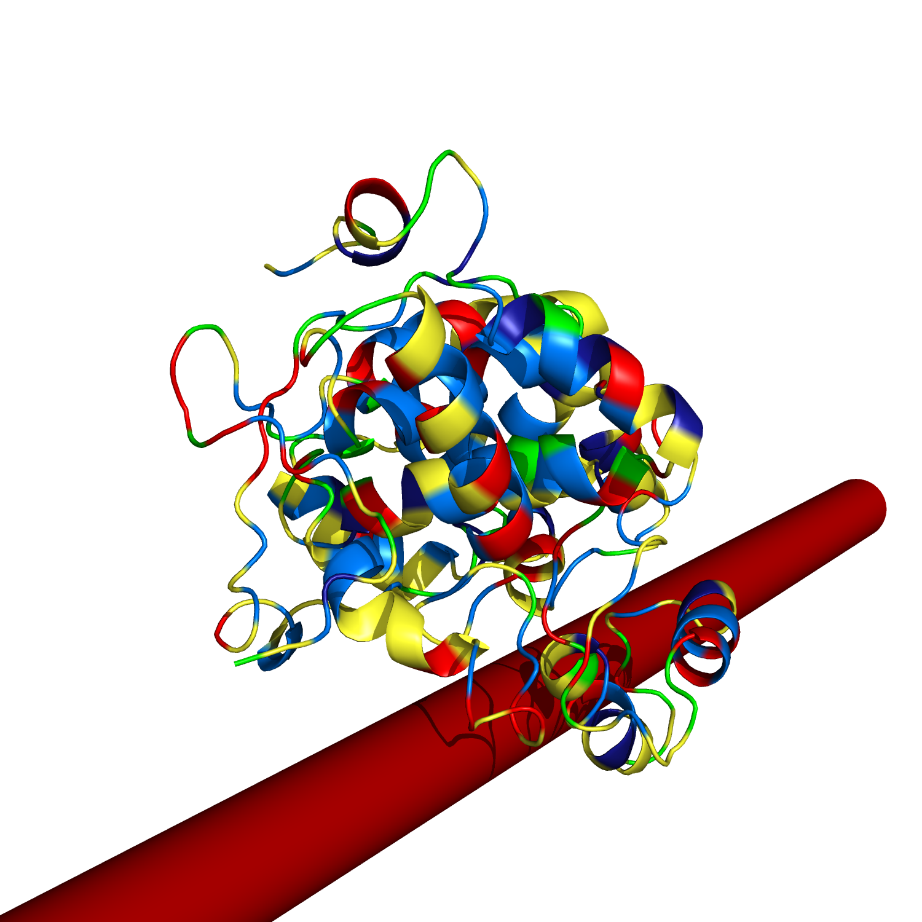
h


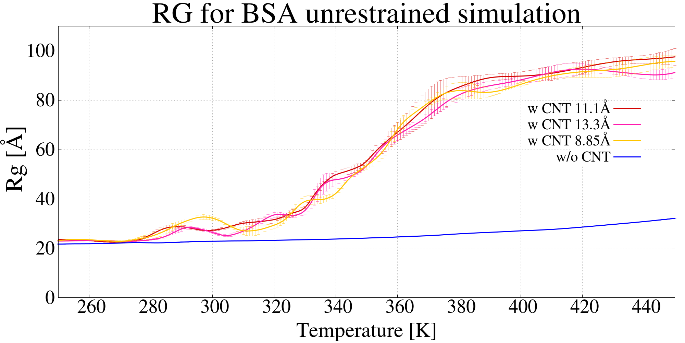
i
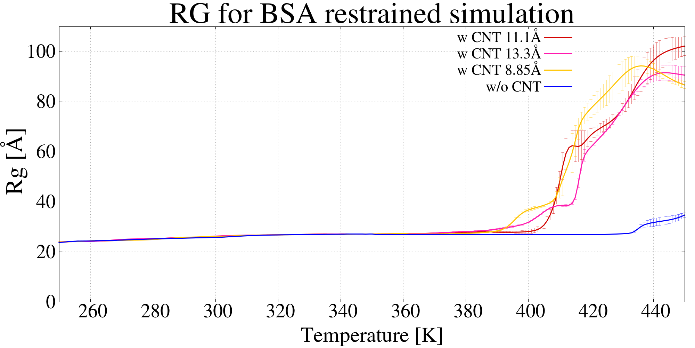
j


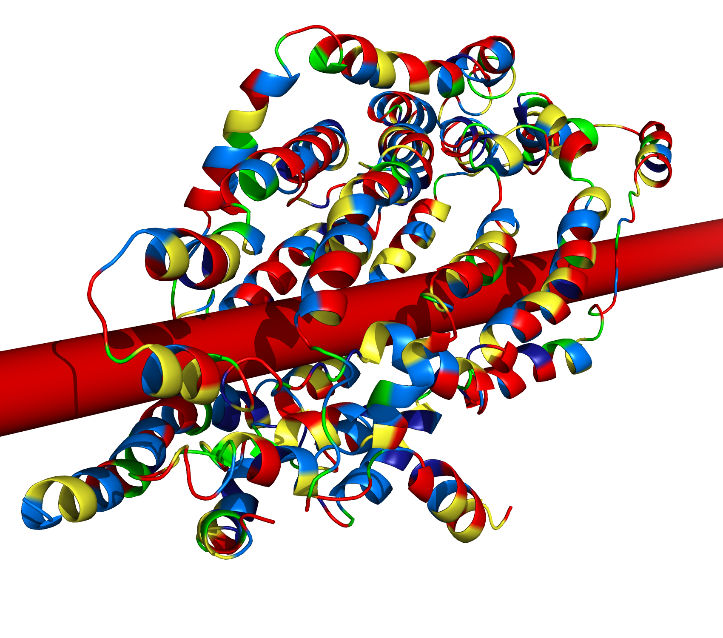
k
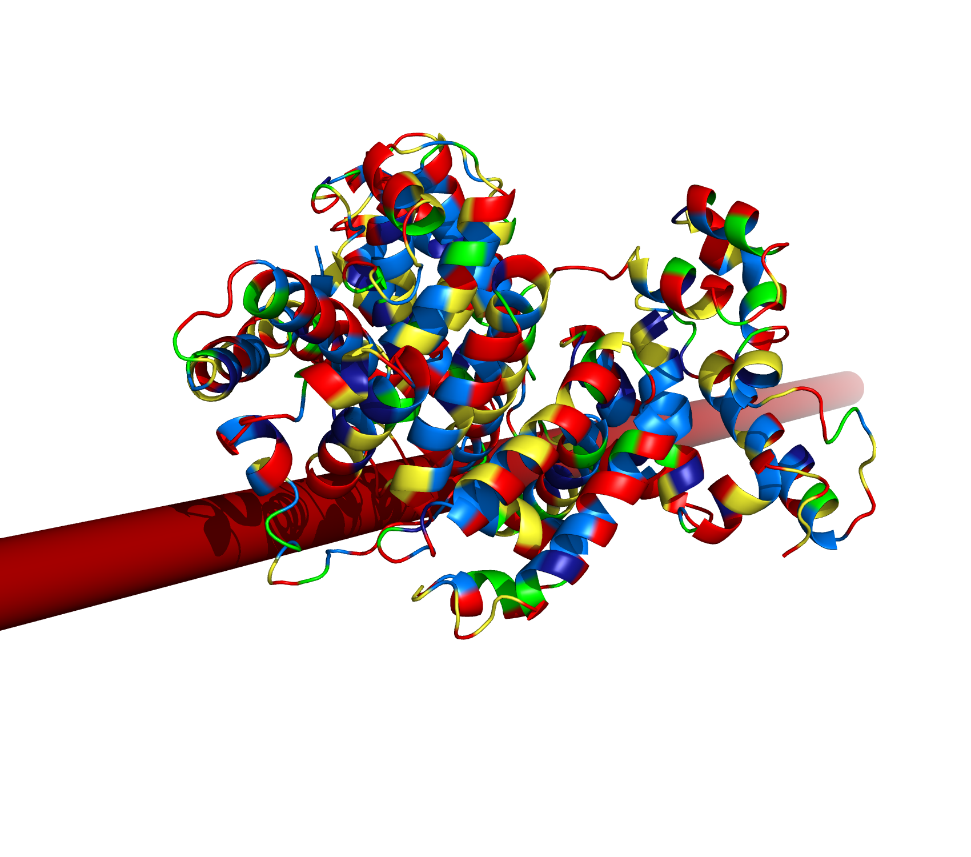
l

**Figure S1**. Radius of gyration (RG) plots for: CT, SBP, and BSA proteins in unrestrained simulations (a, e, i, respectively) and CT, SBP, and BSA proteins in restrained simulations (b, f, and j, respectively). The cartoon representation of the average structure of the CT, SBP and BSA in unrestrained simulations (c, g, k, respectively) an CT, SBP and BSA in restrained simulation (d, h, l, respectively). Different colors of proteins indicate types of the amino-acid residues: hydrophobic (light blue), aromatic (dark blue), polar (yellow), charged (red), cysteine, proline, and glycine (green).


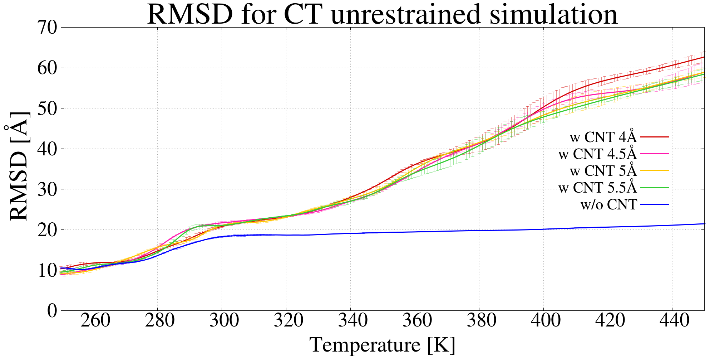
a
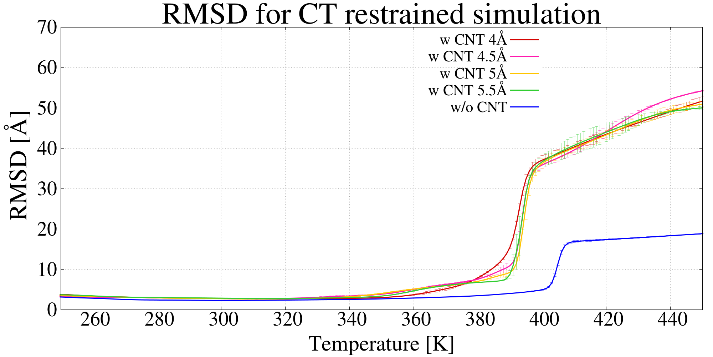
b


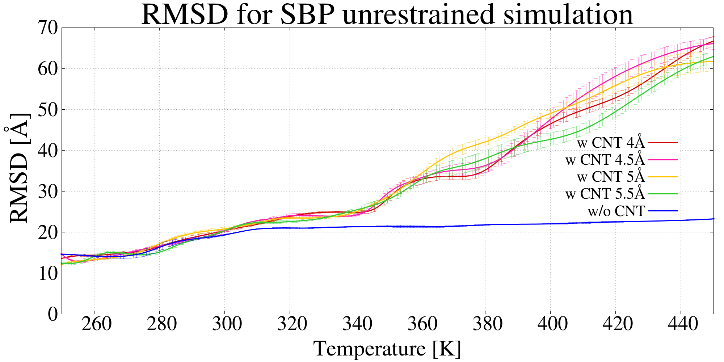
c
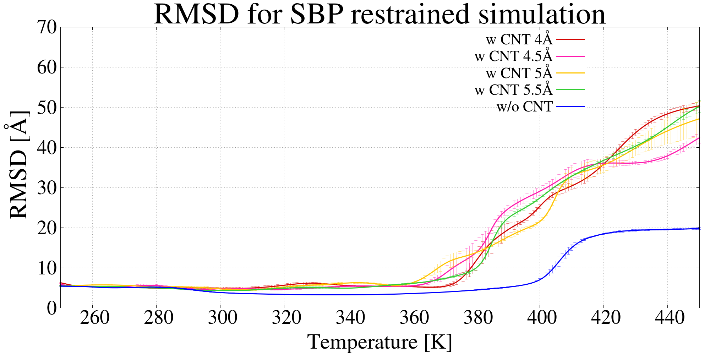
d


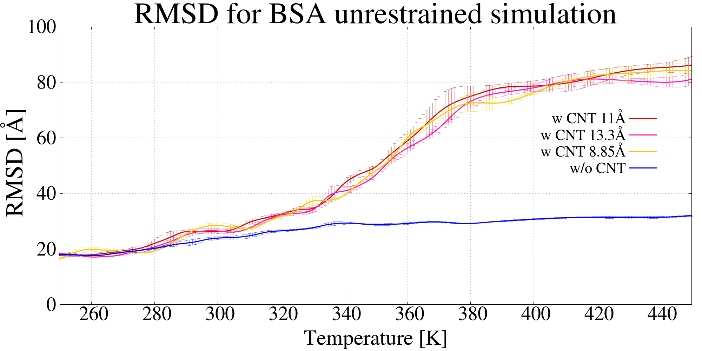
e
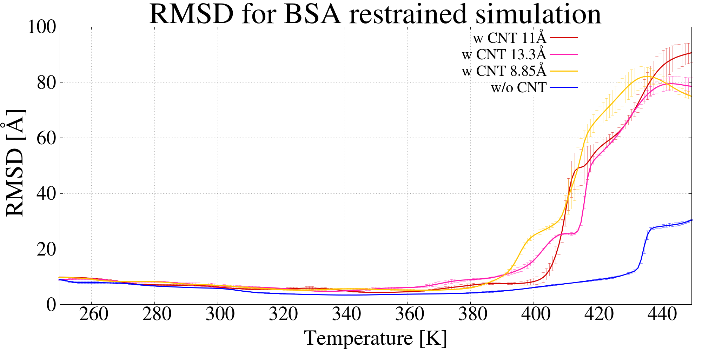
f

**Figure S2**. Root-square mean deviation (RMSD) between the native structure and structures from the simulation averaged for a given temperature, for CT, SBP, and BSA proteins in unrestrained simulations (a, c, e, respectively) and CT, SBP, and BSA proteins in restrained simulations (b, d, and f, respectively).


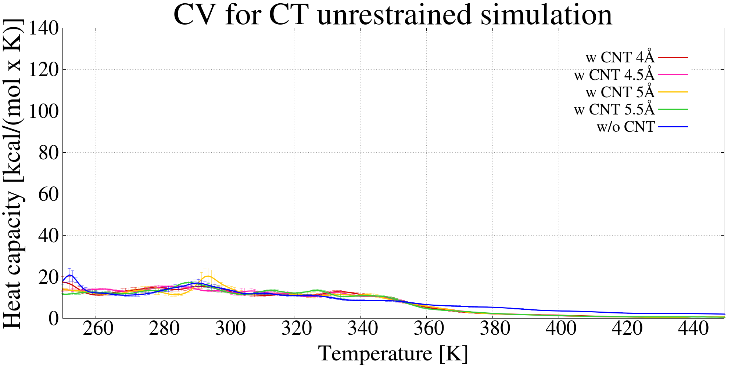

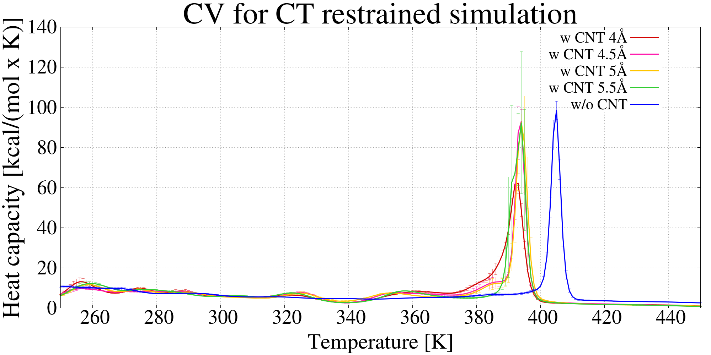
ab


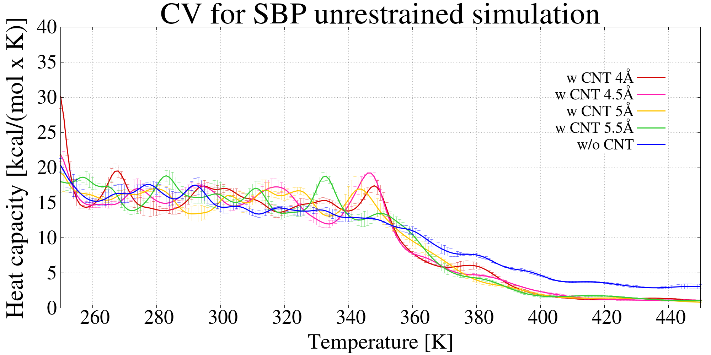
c
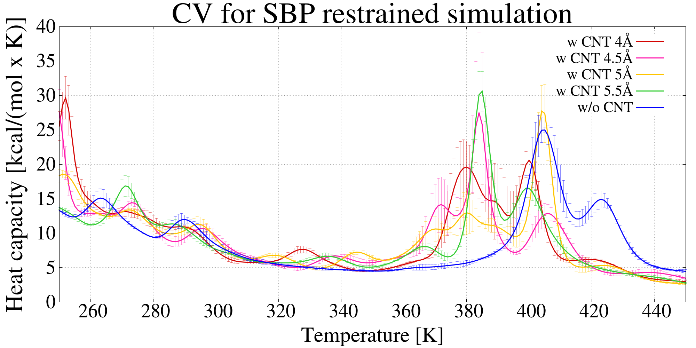
d


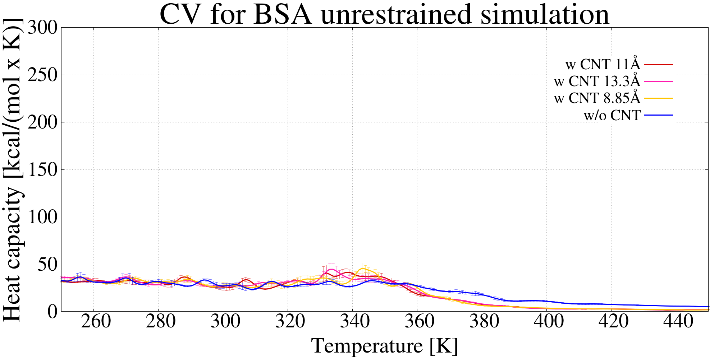
e
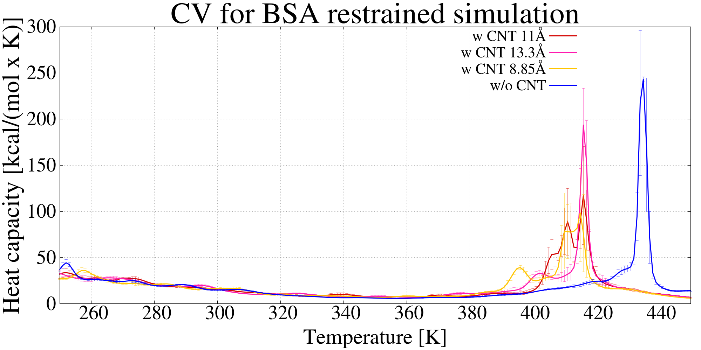
f

**Figure S3**. The heat capacity (CV) plots for CT, SBP, and BSA proteins in unrestrained simulations (a, c, e, respectively) and CT, SBP, and BSA proteins in restrained simulations (b, d, and f, respectively). Blue line represents simulations without nanotube. Red, orange, yellow and green for CT and BSA indicate that simulations were performed with nanotube with radius 4.0, 4.5, 5.0 and 5.5 respectively. Red, orange, yellow for BSA indicate that simulations were performed with nanotube with radius 8.85, 11.1 and 13.3 respectively.


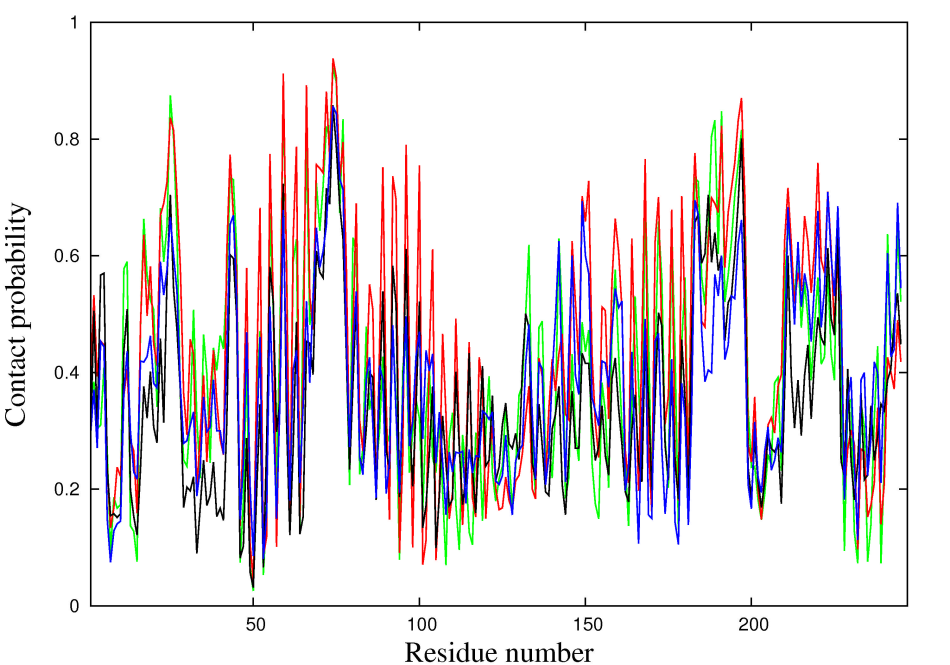
a


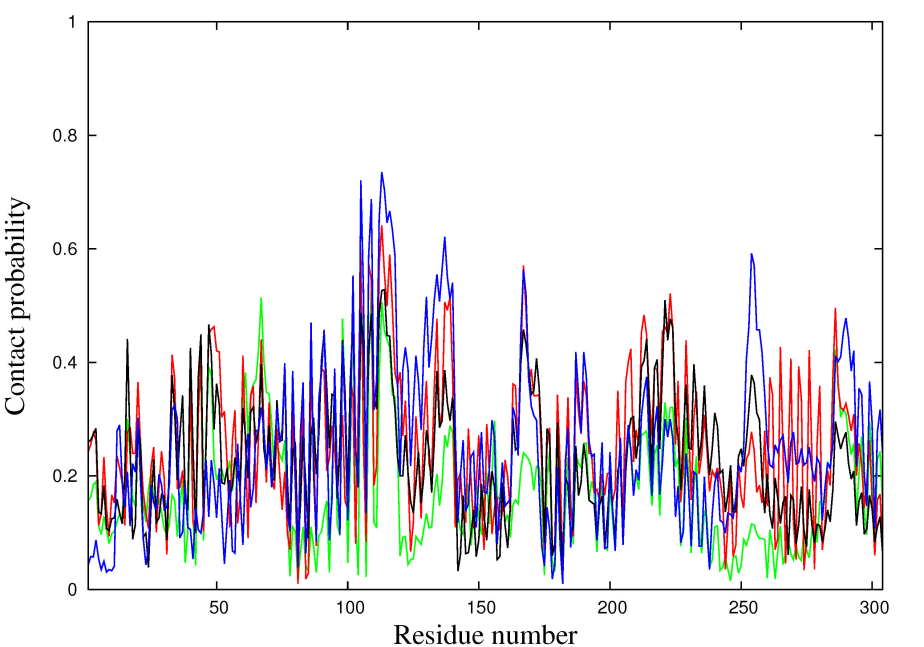
b


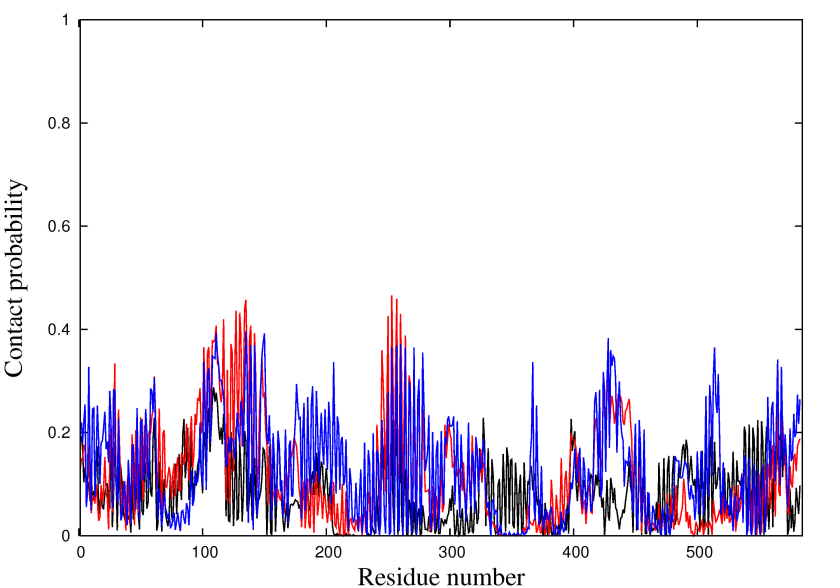
c

**Figure S4**. The probability of the residue to form a contact with CNT against residue number (equation 5) for unrestrained simulations of: a) CT protein with CNT diameters of 4.0Å (red), 4.5Å (green), 5.0Å (blue) and 5.5Å (black); b) SBP protein with CNT diameters of 4.0Å (red), 4.5Å (green), 5.0Å (blue) and 5.5Å (black); c) BSA protein with CNT diameters of 8.85Å (red), 11.1Å (blue), and 13.0Å (black).


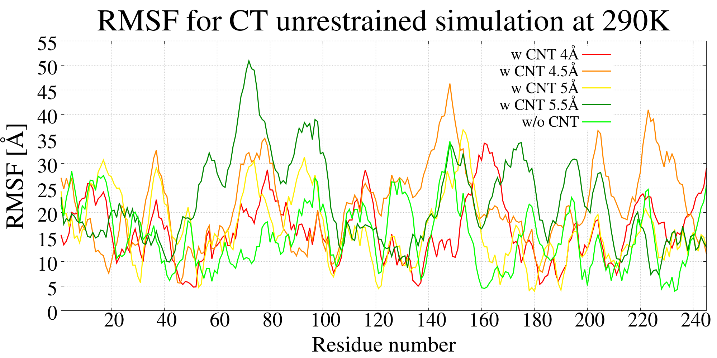
a
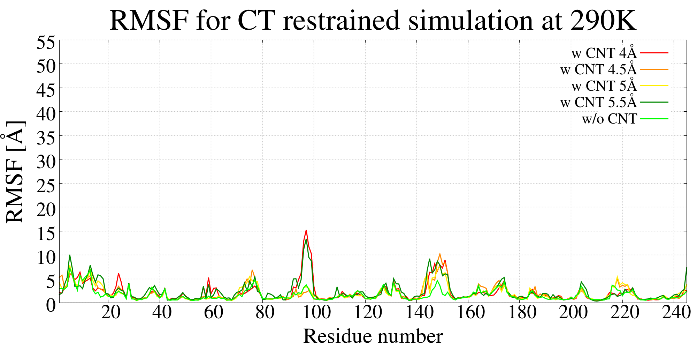
b


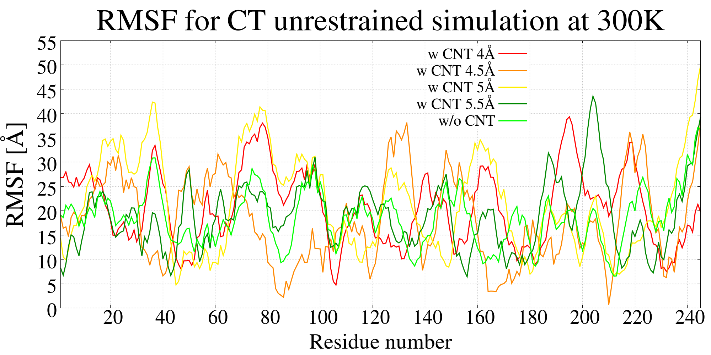
c
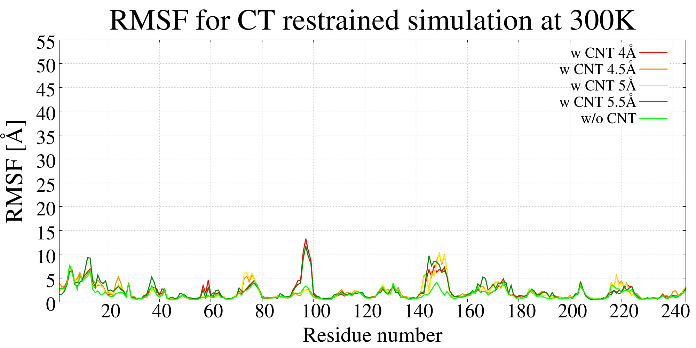
d


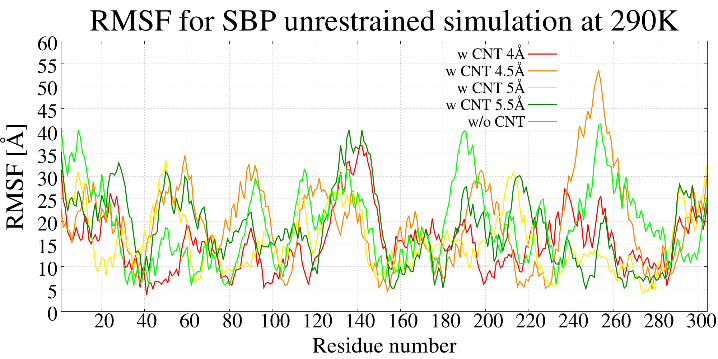
e
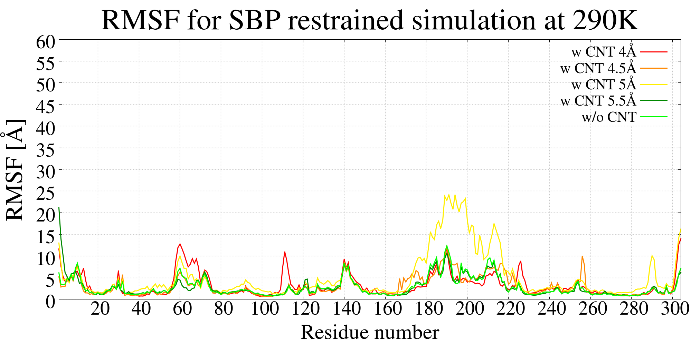
f


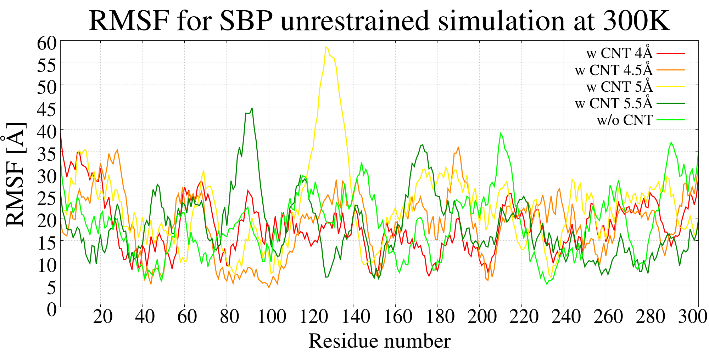
g
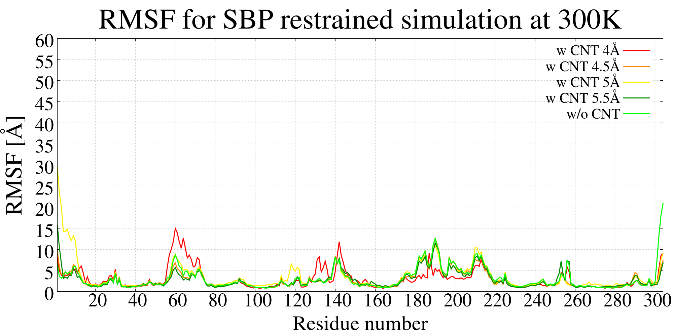
h


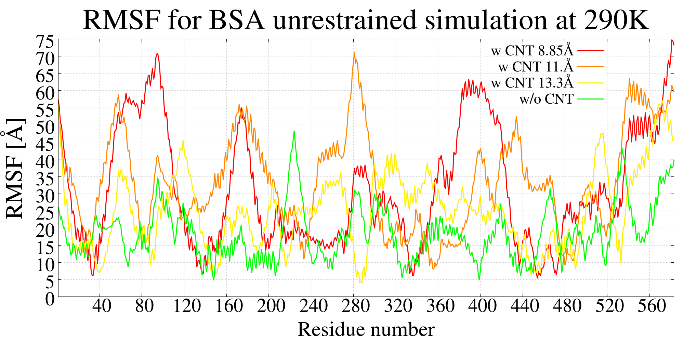
i
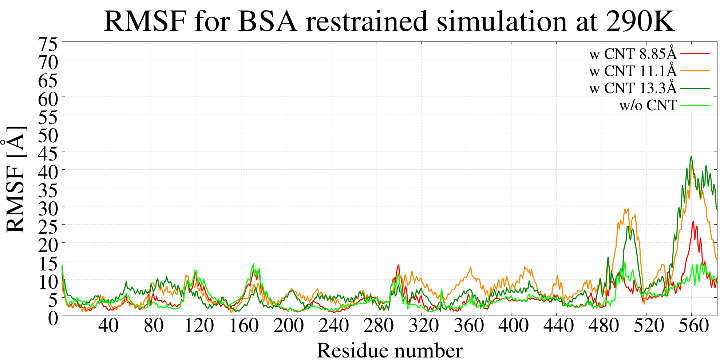
j


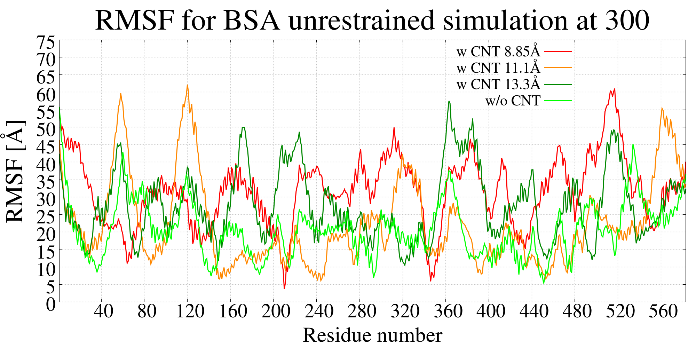
k
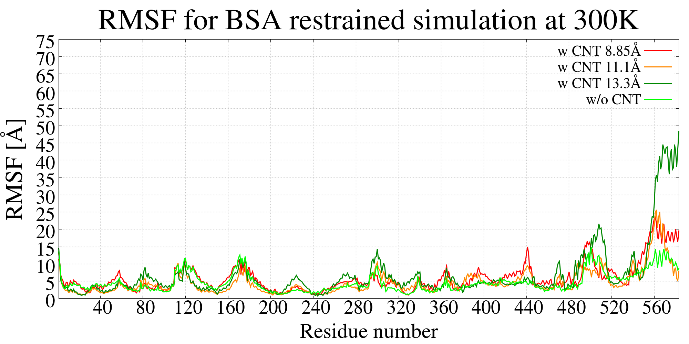
l

**Figure S5**. The RMSF plots obtained from dominant cluster for CT in unrestrained simulations at 290 and 300K (a and c, respectively), and in restrained simulations for 290 and 300K (b and d, respectively; for SBP in unrestrained simulations at 290 and 300K (e and g, respectively), and in restrained simulations at 290 and 300K (f and h, respectively); for BSA in unrestrained simulations at 290 and 300K (m and o, respectively), and in restrained simulations at 290 and 300K (j and l, respectively).

Table S1

Numbers of structures in the families of conformations obtained by minimum-variance clustering of the conformational ensembles of the protein-CT systems resulting from restrained MREMD simulations at various temperatures.

| Nanotube diameter | T [K] | Family index | Number of conformations within a family | Root-mean-square deviation (RMSD) [Å] |
| --- | --- | --- | --- | --- |
| -I | 290 | 1 | 232 | 2.08 |
|  |  | 2 | 179 | 2.11 |
|  |  | 3 | 145 | 2.92 |
|  |  | 4 | 80 | 2.37 |
|  |  | 5 | 68 | 2.60 |
|  |  | 6 | 75 | 2.69 |
|  |  | 7 | 51 | 3.20 |
|  |  | 8 | 51 | 2.17 |
|  |  | 9 | 18 | 2.73 |
|  |  | 10 | 18 | 2.43 |
|  | 300 | 1 | 282 | 2.05 |
|  |  | 2 | 135 | 2.12 |
|  |  | 3 | 105 | 2.58 |
|  |  | 4 | 100 | 2.27 |
|  |  | 5 | 109 | 2.89 |
|  |  | 6 | 72 | 3.23 |
|  |  | 7 | 55 | 2.31 |
|  |  | 8 | 51 | 2.41 |
|  |  | 9 | 45 | 2.62 |
|  |  | 10 | 24 | 2.45 |
| 4Å | 290 | 1 | 57 | 2.98 |
|  |  | 2 | 44 | 2.31 |
|  |  | 3 | 30 | 2.76 |
|  |  | 4 | 11 | 3.34 |
|  | 300 | 1 | 83 | 2.93 |
|  |  | 2 | 47 | 2.73 |
|  |  | 3 | 47 | 2.43 |
|  |  | 4 | 30 | 2.24 |
|  |  | 5 | 15 | 3.25 |
| 4.5Å | 290 | 1 | 64 | 2.65 |
|  |  | 2 | 30 | 2.98 |
|  |  | 3 | 23 | 2.10 |
|  |  | 4 | 13 | 3.07 |
|  | 300 | 1 | 71 | 2.64 |
|  |  | 2 | 42 | 2.12 |
|  |  | 3 | 51 | 3.02 |
|  |  | 4 | 22 | 3.09 |
|  |  | 5 | 20 | 2.89 |
| 5Å | 290 | 1 | 59 | 2.60 |
|  |  | 2 | 39 | 2.82 |
|  |  | 3 | 21 | 2.93 |
|  |  | 4 | 14 | 3.29 |
|  |  | 5 | 12 | 3.41 |
|  | 300 | 1 | 106 | 2.66 |
|  |  | 2 | 39 | 2.95 |
|  |  | 3 | 30 | 2.28 |
|  |  | 4 | 21 | 3.27 |
|  |  | 5 | 29 | 2.85 |
|  |  | 6 | 9 | 3.34 |
| 5.5Å | 290 | 1 | 38 | 3.04 |
|  |  | 2 | 32 | 3.28 |
|  |  | 3 | 26 | 2.79 |
|  |  | 4 | 14 | 3.46 |
|  |  | 5 | 17 | 2.55 |
|  | 300 | 1 | 53 | 2.99 |
|  |  | 2 | 53 | 2.74 |
|  |  | 3 | 37 | 3.26 |
|  |  | 4 | 34 | 2.50 |
|  |  | 5 | 24 | 3.31 |
|  |  | 6 | 11 | 3.03 |

Table S2

Numbers of structures in the families of conformations obtained by minimum-variance clustering of the conformational ensembles of the protein-CT systems resulting from unrestrained MREMD simulations at various temperatures.

| Nanotube diameter | Temp. | Family index | No. of conformations within family | Root-mean-square deviation (RMSD) [Å] |
| --- | --- | --- | --- | --- |
| -I | 290 | 1 | 81 | 16.56 |
|  |  | 2 | 79 | 15.19 |
|  |  | 3 | 73 | 17.53 |
|  |  | 4 | 63 | 17.05 |
|  |  | 5 | 68 | 16.09 |
|  |  | 6 | 63 | 19.24 |
|  |  | 7 | 59 | 11.60 |
|  |  | 8 | 29 | 18.10 |
|  |  | 9 | 47 | 11.97 |
|  |  | 10 | 33 | 18.36 |
|  | 300 | 1 | 77 | 19.24 |
|  |  | 2 | 77 | 19.34 |
|  |  | 3 | 99 | 18.61 |
|  |  | 4 | 76 | 18.31 |
|  |  | 5 | 82 | 19.50 |
|  |  | 6 | 72 | 17.63 |
|  |  | 7 | 58 | 19.74 |
|  |  | 8 | 41 | 18.64 |
|  |  | 9 | 28 | 15.38 |
|  |  | 10 | 31 | 16.66 |
| 4Å | 290 | 1 | 83 | 17.68 |
|  |  | 2 | 74 | 24.59 |
|  |  | 3 | 77 | 19.65 |
|  |  | 4 | 72 | 18.07 |
|  |  | 5 | 48 | 16.49 |
|  |  | 6 | 45 | 13.80 |
|  |  | 7 | 57 | 22.06 |
|  |  | 8 | 38 | 11.28 |
|  |  | 9 | 49 | 19.59 |
|  |  | 10 | 31 | 22.08 |
|  |  | 11 | 25 | 19.61 |
|  |  | 12 | 23 | 16.29 |
|  | 300 | 1 | 83 | 22.35 |
|  |  | 2 | 85 | 22.24 |
|  |  | 3 | 77 | 20.17 |
|  |  | 4 | 68 | 21.39 |
|  |  | 5 | 71 | 19.42 |
|  |  | 6 | 65 | 25.25 |
|  |  | 7 | 43 | 24.38 |
|  |  | 8 | 84 | 18.37 |
|  |  | 9 | 37 | 21.88 |
|  |  | 10 | 35 | 17.75 |
| 4.5Å | 290 | 1 | 82 | 22.94 |
|  |  | 2 | 71 | 20.82 |
|  |  | 3 | 88 | 20.66 |
|  |  | 4 | 72 | 20.11 |
|  |  | 5 | 73 | 19.72 |
|  |  | 6 | 61 | 19.45 |
|  |  | 7 | 57 | 16.66 |
|  |  | 8 | 41 | 12.29 |
|  |  | 9 | 41 | 21.49 |
|  |  | 10 | 19 | 22.29 |
|  | 300 | 1 | 83 | 20.03 |
|  |  | 2 | 74 | 23.04 |
|  |  | 3 | 73 | 19.27 |
|  |  | 4 | 97 | 20.40 |
|  |  | 5 | 58 | 20.61 |
|  |  | 6 | 59 | 24.32 |
|  |  | 7 | 44 | 21.02 |
|  |  | 8 | 55 | 21.00 |
|  |  | 9 | 48 | 23.55 |
|  |  | 10 | 43 | 22.01 |
|  |  | 11 | 22 | 24.45 |
| 5Å | 290 | 1 | 74 | 18.35 |
|  |  | 2 | 75 | 18.60 |
|  |  | 3 | 76 | 14.69 |
|  |  | 4 | 62 | 23.88 |
|  |  | 5 | 77 | 20.41 |
|  |  | 6 | 55 | 15.51 |
|  |  | 7 | 60 | 14.99 |
|  |  | 8 | 48 | 23.99 |
|  |  | 9 | 40 | 12.43 |
|  |  | 10 | 40 | 16.45 |
|  |  | 11 | 28 | 22.97 |
|  | 300 | 1 | 82 | 23.52 |
|  |  | 2 | 72 | 15.57 |
|  |  | 3 | 70 | 24.38 |
|  |  | 4 | 76 | 22.33 |
|  |  | 5 | 59 | 18.65 |
|  |  | 6 | 60 | 23.22 |
|  |  | 7 | 46 | 20.94 |
|  |  | 8 | 73 | 16.36 |
|  |  | 9 | 42 | 18.40 |
|  |  | 10 | 43 | 22.30 |
|  |  | 11 | 30 | 24.97 |
| 5.5Å | 290 | 1 | 82 | 23.34 |
|  |  | 2 | 83 | 23.17 |
|  |  | 3 | 81 | 18.08 |
|  |  | 4 | 79 | 23.03 |
|  |  | 5 | 47 | 14.77 |
|  |  | 6 | 43 | 19.98 |
|  |  | 7 | 27 | 16.20 |
|  |  | 8 | 34 | 20.88 |
|  |  | 9 | 22 | 20.61 |
|  |  | 10 | 48 | 11.91 |
|  |  | 11 | 19 | 18.18 |
|  |  | 12 | 16 | 20.05 |
|  | 300 | 1 | 82 | 20.06 |
|  |  | 2 | 81 | 22.19 |
|  |  | 3 | 85 | 19.22 |
|  |  | 4 | 77 | 22.91 |
|  |  | 5 | 61 | 20.03 |
|  |  | 6 | 64 | 22.02 |
|  |  | 7 | 61 | 23.31 |
|  |  | 8 | 69 | 23.10 |
|  |  | 9 | 32 | 23.81 |
|  |  | 10 | 21 | 23.27 |

Table S3

Numbers of structures in the families of conformations obtained by minimum-variance clustering of the conformational ensembles of the protein-SBP systems resulting from restrained MREMD simulations at various temperatures.

| Nanotube diameter | Temp. | Family index | No. of conformations within family | Root-mean-square deviation (RMSD) [Å] |
| --- | --- | --- | --- | --- |
| -I | 290 | 1 | 197 | 3.52 |
|  |  | 2 | 146 | 3.95 |
|  |  | 3 | 112 | 7.14 |
|  |  | 4 | 54 | 6.80 |
|  |  | 5 | 53 | 4.62 |
|  |  | 6 | 71 | 4.05 |
|  |  | 7 | 65 | 4.48 |
|  |  | 8 | 52 | 4.24 |
|  |  | 9 | 42 | 3.87 |
|  |  | 10 | 39 | 7.22 |
|  | 300 | 1 | 197 | 3.52 |
|  |  | 2 | 146 | 3.95 |
|  |  | 3 | 112 | 7.14 |
|  |  | 4 | 54 | 6.80 |
|  |  | 5 | 53 | 4.62 |
|  |  | 6 | 71 | 4.05 |
|  |  | 7 | 65 | 4.48 |
|  |  | 8 | 52 | 4.24 |
|  |  | 9 | 42 | 3.87 |
|  |  | 10 | 39 | 7.22 |
| 4Å | 290 | 1 | 78 | 4.04 |
|  |  | 2 | 109 | 3.85 |
|  |  | 3 | 68 | 8.51 |
|  |  | 4 | 67 | 4.14 |
|  |  | 5 | 46 | 3.70 |
|  |  | 6 | 86 | 4.22 |
|  |  | 7 | 52 | 7.76 |
|  |  | 8 | 35 | 4.00 |
|  |  | 9 | 39 | 6.80 |
|  |  | 10 | 64 | 3.93 |
|  |  | 11 | 30 | 5.55 |
|  |  | 12 | 33 | 4.16 |
|  |  | 13 | 25 | 4.45 |
|  |  | 14 | 26 | 7.76 |
|  |  | 15 | 8 | 5.18 |
|  |  | 16 | 20 | 7.96 |
|  |  | 17 | 14 | 10.92 |
|  |  | 18 | 4 | 9.08 |
|  | 300 | 1 | 146 | 3.84 |
|  |  | 2 | 96 | 3.57 |
|  |  | 3 | 92 | 3.78 |
|  |  | 4 | 100 | 4.19 |
|  |  | 5 | 69 | 8.51 |
|  |  | 6 | 62 | 4.11 |
|  |  | 7 | 66 | 3.79 |
|  |  | 8 | 50 | 7.98 |
|  |  | 9 | 50 | 7.11 |
|  |  | 10 | 41 | 11.11 |
|  |  | 11 | 31 | 9.11 |
|  |  | 12 | 38 | 3.95 |
|  |  | 13 | 24 | 5.29 |
|  |  | 14 | 17 | 8.05 |
|  |  | 15 | 9 | 10.10 |
|  |  | 16 | 5 | 5.22 |
| 4.5Å | 290 | 1 | 106 | 3.72 |
|  |  | 2 | 81 | 4.05 |
|  |  | 3 | 74 | 4.44 |
|  |  | 4 | 90 | 4.15 |
|  |  | 5 | 52 | 4.43 |
|  |  | 6 | 62 | 4.51 |
|  |  | 7 | 59 | 4.54 |
|  |  | 8 | 62 | 4.27 |
|  |  | 9 | 86 | 3.59 |
|  |  | 10 | 34 | 11.25 |
|  |  | 11 | 25 | 4.16 |
|  |  | 12 | 20 | 11.46 |
|  |  | 13 | 14 | 11.32 |
|  |  | 14 | 6 | 7.94 |
|  | 300 | 1 | 189 | 3.49 |
|  |  | 2 | 85 | 4.34 |
|  |  | 3 | 106 | 3.51 |
|  |  | 4 | 85 | 3.83 |
|  |  | 5 | 69 | 11.34 |
|  |  | 6 | 50 | 4.02 |
|  |  | 7 | 72 | 4.04 |
|  |  | 8 | 66 | 3.55 |
|  |  | 9 | 48 | 4.15 |
|  |  | 10 | 28 | 4.43 |
|  |  | 11 | 32 | 4.44 |
|  |  | 12 | 18 | 9.02 |
|  |  | 13 | 10 | 11.25 |
|  |  | 14 | 7 | 8.06 |
|  |  | 15 | 10 | 4.71 |
| 5Å | 290 | 1 | 94 | 7.11 |
|  |  | 2 | 82 | 4.57 |
|  |  | 3 | 95 | 5.12 |
|  |  | 4 | 66 | 7.62 |
|  |  | 5 | 78 | 4.52 |
|  |  | 6 | 55 | 4.64 |
|  |  | 7 | 63 | 5.02 |
|  |  | 8 | 41 | 4.95 |
|  |  | 9 | 47 | 5.01 |
|  |  | 10 | 37 | 7.56 |
|  |  | 11 | 16 | 7.97 |
|  |  | 12 | 34 | 4.22 |
|  |  | 13 | 18 | 4.45 |
|  |  | 14 | 18 | 3.80 |
|  |  | 15 | 20 | 4.10 |
|  | 300 | 1 | 103 | 4.79 |
|  |  | 2 | 73 | 4.02 |
|  |  | 3 | 71 | 4.51 |
|  |  | 4 | 60 | 7.50 |
|  |  | 5 | 64 | 4.59 |
|  |  | 6 | 63 | 3.47 |
|  |  | 7 | 73 | 7.02 |
|  |  | 8 | 73 | 3.91 |
|  |  | 9 | 70 | 4.66 |
|  |  | 10 | 56 | 3.66 |
|  |  | 11 | 20 | 6.92 |
|  |  | 12 | 19 | 8.04 |
|  |  | 13 | 28 | 3.85 |
|  |  | 14 | 23 | 7.56 |
|  |  | 15 | 17 | 5.06 |
|  |  | 16 | 16 | 4.58 |
|  |  | 17 | 4 | 10.19 |
| 5.,5Å | 290 | 1 | 82 | 3.66 |
|  |  | 2 | 59 | 4.04 |
|  |  | 3 | 67 | 4.22 |
|  |  | 4 | 68 | 4.85 |
|  |  | 5 | 94 | 4.00 |
|  |  | 6 | 39 | 4.00 |
|  |  | 7 | 36 | 4.07 |
|  |  | 8 | 51 | 5.80 |
|  |  | 9 | 29 | 4.39 |
|  |  | 10 | 54 | 7.45 |
|  |  | 11 | 52 | 3.60 |
|  |  | 12 | 59 | 4.62 |
|  |  | 13 | 32 | 4.65 |
|  |  | 14 | 24 | 4.78 |
|  |  | 15 | 23 | 4.36 |
|  |  | 16 | 18 | 5.05 |
|  |  | 17 | 3 | 7.74 |
|  | 300 | 1 | 116 | 3.54 |
|  |  | 2 | 82 | 4.80 |
|  |  | 3 | 76 | 3.87 |
|  |  | 4 | 89 | 3.56 |
|  |  | 5 | 59 | 5.75 |
|  |  | 6 | 67 | 4.06 |
|  |  | 7 | 57 | 4.74 |
|  |  | 8 | 56 | 3.68 |
|  |  | 9 | 46 | 3.94 |
|  |  | 10 | 36 | 4.05 |
|  |  | 11 | 46 | 3.37 |
|  |  | 12 | 35 | 3.92 |
|  |  | 13 | 18 | 5.65 |
|  |  | 14 | 32 | 7.64 |
|  |  | 15 | 15 | 5.52 |
|  |  | 16 | 24 | 4.16 |
|  |  | 17 | 19 | 5.05 |
|  |  | 18 | 10 | 10.54 |
|  |  | 19 | 2 | 10.41 |

Table S4

Numbers of structures in the families of conformations obtained by minimum-variance clustering of the conformational ensembles of the protein-SBP systems resulting from unrestrained MREMD simulations at various temperatures.

| Nanotube diameter | Temp. | Family index | No. of conformations within family | Root-mean-square deviation (RMSD) [Å] |
| --- | --- | --- | --- | --- |
| -I | 290 | 1 | 77 | 21.16 |
|  |  | 2 | 75 | 15.42 |
|  |  | 3 | 67 | 20.05 |
|  |  | 4 | 67 | 17.82 |
|  |  | 5 | 58 | 20.02 |
|  |  | 6 | 59 | 17.94 |
|  |  | 7 | 58 | 19.12 |
|  |  | 8 | 62 | 19.54 |
|  |  | 9 | 70 | 18.55 |
|  |  | 10 | 45 | 15.99 |
|  | 300 | 1 | 75 | 20.05 |
|  |  | 2 | 79 | 18.57 |
|  |  | 3 | 70 | 17.86 |
|  |  | 4 | 65 | 20.55 |
|  |  | 5 | 61 | 20.32 |
|  |  | 6 | 55 | 21.63 |
|  |  | 7 | 37 | 20.14 |
|  |  | 8 | 54 | 20.81 |
|  |  | 9 | 39 | 18.03 |
|  |  | 10 | 75 | 19.44 |
| 4Å | 290 | 1 | 79 | 16.84 |
|  |  | 2 | 73 | 21.55 |
|  |  | 3 | 72 | 20.80 |
|  |  | 4 | 87 | 17.83 |
|  |  | 5 | 67 | 20.96 |
|  |  | 6 | 61 | 20.38 |
|  |  | 7 | 61 | 22.75 |
|  |  | 8 | 44 | 17.56 |
|  |  | 9 | 32 | 14.89 |
|  |  | 10 | 19 | 22.73 |
|  |  | 11 | 24 | 19.41 |
|  | 300 | 1 | 96 | 19.23 |
|  |  | 2 | 77 | 19.56 |
|  |  | 3 | 75 | 22.80 |
|  |  | 4 | 73 | 22.40 |
|  |  | 5 | 70 | 24.35 |
|  |  | 6 | 79 | 20.53 |
|  |  | 7 | 45 | 17.10 |
|  |  | 8 | 29 | 20.77 |
|  |  | 9 | 39 | 22.44 |
| 4.5Å | 290 | 1 | 67 | 22.47 |
|  |  | 2 | 63 | 19.50 |
|  |  | 3 | 66 | 19.87 |
|  |  | 4 | 71 | 15.78 |
|  |  | 5 | 65 | 14.84 |
|  |  | 6 | 60 | 15.78 |
|  |  | 7 | 68 | 18.16 |
|  |  | 8 | 40 | 14.93 |
|  |  | 9 | 48 | 21.20 |
|  |  | 10 | 58 | 18.50 |
|  | 300 | 1 | 79 | 20.01 |
|  |  | 2 | 78 | 21.32 |
|  |  | 3 | 80 | 17.50 |
|  |  | 4 | 65 | 15.87 |
|  |  | 5 | 62 | 23.56 |
|  |  | 6 | 66 | 19.08 |
|  |  | 7 | 36 | 26.41 |
|  |  | 8 | 36 | 21.84 |
|  |  | 9 | 28 | 20.01 |
|  |  | 10 | 38 | 17.57 |
|  |  | 11 | 21 | 23.76 |
| 5Å | 290 | 1 | 58 | 17.56 |
|  |  | 2 | 55 | 21.94 |
|  |  | 3 | 56 | 20.07 |
|  |  | 4 | 52 | 18.49 |
|  |  | 5 | 56 | 22.69 |
|  |  | 6 | 44 | 22.24 |
|  |  | 7 | 57 | 18.72 |
|  |  | 8 | 47 | 22.11 |
|  |  | 9 | 37 | 18.10 |
|  | 300 | 1 | 54 | 24.17 |
|  |  | 2 | 60 | 21.98 |
|  |  | 3 | 57 | 18.97 |
|  |  | 4 | 51 | 22.31 |
|  |  | 5 | 50 | 23.03 |
|  |  | 6 | 64 | 20.52 |
|  |  | 7 | 53 | 23.18 |
|  |  | 8 | 36 | 21.33 |
|  |  | 9 | 33 | 20.80 |
|  |  | 10 | 25 | 17.95 |
| 5.5Å | 290 | 1 | 78 | 19.85 |
|  |  | 2 | 83 | 14.92 |
|  |  | 3 | 79 | 18.78 |
|  |  | 4 | 71 | 17.46 |
|  |  | 5 | 59 | 19.82 |
|  |  | 6 | 61 | 19.56 |
|  |  | 7 | 42 | 16.38 |
|  |  | 8 | 64 | 16.94 |
|  | 300 | 1 | 74 | 19.44 |
|  |  | 2 | 70 | 20.01 |
|  |  | 3 | 73 | 20.21 |
|  |  | 4 | 65 | 23.85 |
|  |  | 5 | 47 | 17.61 |
|  |  | 6 | 37 | 25.02 |
|  |  | 7 | 47 | 20.22 |
|  |  | 8 | 25 | 18.85 |
|  |  | 9 | 36 | 15.15 |
|  |  | 10 | 56 | 22.50 |
|  |  | 11 | 33 | 18.92 |
|  |  | 12 | 22 | 23.49 |

Table S5

Numbers of structures in the families of conformations obtained by minimum-variance clustering of the conformational ensembles of the protein-BSE systems resulting from restrained MREMD simulations at various temperatures.

| Nanotube diameter | Temp. | Family index | No. of conformations within family | Root-mean-square deviation (RMSD) [Å] |
| --- | --- | --- | --- | --- |
| -I | 290 | 1 | 122 | 5.73 |
|  |  | 2 | 105 | 7.66 |
|  |  | 3 | 108 | 5.23 |
|  |  | 4 | 57 | 7.66 |
|  |  | 5 | 53 | 4.26 |
|  |  | 6 | 35 | 4.90 |
|  |  | 7 | 36 | 3.96 |
|  |  | 8 | 40 | 8.59 |
|  |  | 9 | 27 | 8.49 |
|  |  | 10 | 27 | 7.35 |
|  | 300 | 1 | 102 | 5.47 |
|  |  | 2 | 79 | 3.80 |
|  |  | 3 | 82 | 3.97 |
|  |  | 4 | 60 | 8.34 |
|  |  | 5 | 66 | 5.12 |
|  |  | 6 | 62 | 7.57 |
|  |  | 7 | 58 | 7.50 |
|  |  | 8 | 46 | 6.33 |
|  |  | 9 | 45 | 3.25 |
|  |  | 10 | 25 | 4.78 |
| 8Å | 290 | 1 | 98 | 6.26 |
|  |  | 2 | 75 | 8.90 |
|  |  | 3 | 75 | 7.28 |
|  |  | 4 | 71 | 7.17 |
|  |  | 5 | 67 | 7.77 |
|  |  | 6 | 56 | 9.05 |
|  |  | 7 | 41 | 5.84 |
|  |  | 8 | 36 | 5.07 |
|  |  | 9 | 44 | 7.26 |
|  |  | 10 | 27 | 7.20 |
|  |  | 11 | 14 | 9.04 |
|  |  | 12 | 4 | 10.30 |
|  | 300 | 1 | 77 | 6.98 |
|  |  | 2 | 65 | 7.15 |
|  |  | 3 | 70 | 6.17 |
|  |  | 4 | 44 | 6.94 |
|  |  | 5 | 35 | 7.21 |
|  |  | 6 | 34 | 4.99 |
|  |  | 7 | 38 | 5.95 |
|  |  | 8 | 37 | 11.10 |
|  |  | 9 | 34 | 5.67 |
|  |  | 10 | 38 | 6.25 |
|  |  | 11 | 27 | 9.94 |
|  |  | 12 | 34 | 3.89 |
|  |  | 13 | 24 | 7.21 |
|  |  | 14 | 18 | 4.98 |
|  |  | 15 | 13 | 6.03 |
|  |  | 16 | 19 | 9.00 |
|  |  | 17 | 15 | 7.68 |
|  |  | 18 | 15 | 7.51 |
|  |  | 19 | 11 | 7.49 |
|  |  | 20 | 7 | 7.42 |
| 11Å | 290 | 1 | 81 | 11.13 |
|  |  | 2 | 73 | 6.06 |
|  |  | 3 | 58 | 6.65 |
|  |  | 4 | 38 | 7.00 |
|  |  | 5 | 47 | 5.72 |
|  |  | 6 | 40 | 7.03 |
|  |  | 7 | 52 | 7.56 |
|  |  | 8 | 42 | 6.03 |
|  |  | 9 | 34 | 7.48 |
|  |  | 10 | 41 | 6.47 |
|  |  | 11 | 24 | 6.03 |
|  |  | 12 | 21 | 9.33 |
|  |  | 13 | 15 | 4.70 |
|  |  | 14 | 13 | 5.93 |
|  | 300 | 1 | 117 | 5.93 |
|  |  | 2 | 88 | 6.37 |
|  |  | 3 | 92 | 7.44 |
|  |  | 4 | 72 | 5.76 |
|  |  | 5 | 59 | 5.46 |
|  |  | 6 | 50 | 5.60 |
|  |  | 7 | 27 | 6.69 |
|  |  | 8 | 48 | 11.18 |
|  |  | 9 | 22 | 3.95 |
|  |  | 10 | 27 | 6.84 |
|  |  | 11 | 23 | 6.75 |
| 13Å | 290 | 1 | 78 | 11.19 |
|  |  | 2 | 53 | 5.32 |
|  |  | 3 | 56 | 6.84 |
|  |  | 4 | 42 | 8.72 |
|  |  | 5 | 34 | 8.22 |
|  |  | 6 | 22 | 7.07 |
|  |  | 7 | 35 | 10.64 |
|  |  | 8 | 25 | 9.04 |
|  |  | 9 | 32 | 8.13 |
|  |  | 10 | 20 | 6.51 |
|  |  | 11 | 23 | 8.70 |
|  |  | 12 | 18 | 5.93 |
|  |  | 13 | 25 | 5.18 |
|  |  | 14 | 22 | 8.23 |
|  |  | 15 | 19 | 10.03 |
|  |  | 16 | 16 | 7.03 |
|  |  | 17 | 7 | 7.74 |
|  |  | 18 | 11 | 8.84 |
|  |  | 19 | 11 | 6.75 |
|  |  | 20 | 13 | 10.31 |
|  |  | 21 | 7 | 11.07 |
|  |  | 22 | 5 | 9.78 |
|  |  | 23 | 5 | 7.92 |
|  | 300 | 1 | 51 | 10.17 |
|  |  | 2 | 55 | 7.38 |
|  |  | 3 | 49 | 4.85 |
|  |  | 4 | 40 | 4.13 |
|  |  | 5 | 51 | 6.69 |
|  |  | 6 | 42 | 10.55 |
|  |  | 7 | 48 | 7.20 |
|  |  | 8 | 27 | 8.38 |
|  |  | 9 | 28 | 10.36 |
|  |  | 10 | 45 | 8.18 |
|  |  | 11 | 27 | 9.35 |
|  |  | 12 | 20 | 9.97 |
|  |  | 13 | 35 | 8.49 |
|  |  | 14 | 21 | 8.70 |
|  |  | 15 | 17 | 6.64 |
|  |  | 16 | 16 | 9.21 |
|  |  | 17 | 29 | 6.50 |
|  |  | 18 | 26 | 11.06 |
|  |  | 19 | 12 | 8.28 |
|  |  | 20 | 10 | 5.76 |
|  |  | 21 | 10 | 5.23 |
|  |  | 22 | 8 | 7.21 |
|  |  | 23 | 6 | 7.35 |

Table S6

Numbers of structures in the families of conformations obtained by minimum-variance clustering of the conformational ensembles of the protein-BSE systems resulting from unconstrained MREMD simulations at various temperatures.

| Nanotube diameter | Temp. | Family index | No. of conformations within family | Root-mean-square deviation (RMSD) [Å] |
| --- | --- | --- | --- | --- |
| -I | 290 | 1 | 80 | 20.18 |
|  |  | 2 | 73 | 27.92 |
|  |  | 3 | 68 | 22.58 |
|  |  | 4 | 48 | 31.59 |
|  |  | 5 | 46 | 24.63 |
|  |  | 6 | 28 | 20.91 |
|  |  | 7 | 30 | 20.01 |
|  |  | 8 | 24 | 21.00 |
|  |  | 9 | 26 | 22.22 |
|  |  | 10 | 17 | 21.23 |
|  | 300 | 1 | 78 | 22.23 |
|  |  | 2 | 72 | 21.04 |
|  |  | 3 | 63 | 24.39 |
|  |  | 4 | 52 | 30.62 |
|  |  | 5 | 52 | 23.95 |
|  |  | 6 | 33 | 27.01 |
|  |  | 7 | 25 | 28.43 |
|  |  | 8 | 33 | 25.29 |
|  |  | 9 | 28 | 22.78 |
|  |  | 10 | 13 | 28.75 |
| 8Å | 290 | 1 | 83 | 35.63 |
|  |  | 2 | 79 | 20.62 |
|  |  | 3 | 52 | 19.18 |
|  |  | 4 | 35 | 27.76 |
|  |  | 5 | 38 | 28.84 |
|  |  | 6 | 48 | 28.19 |
|  |  | 7 | 30 | 34.99 |
|  |  | 8 | 31 | 33.32 |
|  |  | 9 | 18 | 23.83 |
|  |  | 10 | 16 | 26.36 |
|  | 300 | 1 | 81 | 33.42 |
|  |  | 2 | 76 | 26.55 |
|  |  | 3 | 76 | 35.36 |
|  |  | 4 | 51 | 24.52 |
|  |  | 5 | 54 | 31.71 |
|  |  | 6 | 54 | 28.47 |
|  |  | 7 | 39 | 24.13 |
|  |  | 8 | 18 | 20.01 |
|  |  | 9 | 21 | 26.32 |
|  |  | 10 | 9 | 35.67 |
| 11Å | 290 | 1 | 61 | 36.85 |
|  |  | 2 | 49 | 28.00 |
|  |  | 3 | 34 | 23.77 |
|  |  | 4 | 38 | 23.34 |
|  |  | 5 | 56 | 19.77 |
|  |  | 6 | 31 | 29.77 |
|  |  | 7 | 41 | 33.82 |
|  |  | 8 | 24 | 25.80 |
|  |  | 9 | 30 | 25.79 |
|  |  | 10 | 27 | 25.40 |
|  |  | 11 | 17 | 25.40 |
|  |  | 12 | 9 | 19.69 |
|  | 300 | 1 | 72 | 25.60 |
|  |  | 2 | 69 | 25.59 |
|  |  | 3 | 66 | 29.73 |
|  |  | 4 | 64 | 25.84 |
|  |  | 5 | 58 | 28.14 |
|  |  | 6 | 40 | 27.29 |
|  |  | 7 | 29 | 34.71 |
|  |  | 8 | 25 | 23.84 |
|  |  | 9 | 5 | 31.17 |
| 13Å | 290 | 1 | 80 | 27.45 |
|  |  | 2 | 74 | 29.72 |
|  |  | 3 | 63 | 23.63 |
|  |  | 4 | 38 | 28.44 |
|  |  | 5 | 42 | 22.28 |
|  |  | 6 | 31 | 32.52 |
|  |  | 7 | 35 | 23.34 |
|  |  | 8 | 12 | 32.54 |
|  |  | 9 | 22 | 29.65 |
|  |  | 10 | 19 | 20.72 |
|  |  | 11 | 22 | 25.19 |
|  | 300 | 1 | 78 | 29.48 |
|  |  | 2 | 74 | 32.70 |
|  |  | 3 | 70 | 28.28 |
|  |  | 4 | 62 | 23.64 |
|  |  | 5 | 35 | 31.81 |
|  |  | 6 | 40 | 25.97 |
|  |  | 7 | 49 | 20.34 |
|  |  | 8 | 33 | 30.31 |
|  |  | 9 | 14 | 21.88 |
|  |  | 10 | 18 | 27.70 |
